# Genome-Wide Atlas of Promoter Expression Reveals Contribution of Transcribed Regulatory Elements to Genetic Control of Disuse-Mediated Atrophy of Skeletal Muscle

**DOI:** 10.3390/biology10060557

**Published:** 2021-06-20

**Authors:** Sergey S. Pintus, Ilya R. Akberdin, Ivan Yevshin, Pavel Makhnovskii, Oksana Tyapkina, Islam Nigmetzyanov, Leniz Nurullin, Ruslan Devyatiyarov, Elena Shagimardanova, Daniil Popov, Fedor A. Kolpakov, Oleg Gusev, Guzel R. Gazizova

**Affiliations:** 1 Laboratory of Bioinformatics, Federal Research Center for Information and Computational Technologies, 630090 Novosibirsk, Russia; 2 Department of Computational Biology, Scientific Center for Information Technologies and Artificial Intelligence, Sirius University of Science and Technology, 354340 Sochi, Russia; 3 BIOSOFT.RU LLC, 630058 Novosibirsk, Russia; sspintus@biosoft.ru (S.S.P.); akberdinir@biosoft.ru (I.R.A.); ivan@biosoft.ru (I.Y.); 4 Faculty of Natural Sciences, Novosibirsk State University, 630090 Novosibirsk, Russia; 5 Institute of Biomedical Problems of the Russian Academy of Sciences, Moscow 123007, Russia; maxpauel@gmail.com (P.M.); danil-popov@yandex.ru (D.P.); 6 Kazan Institute of Biochemistry and Biophysics FRC Kazan Scientific Center of RAS, 420007 Kazan, Russia; anti-toxin@mail.ru (O.T.); leniz2001@mail.ru (L.N.); 7 Department of Biology, Kazan State Medical University, 420012 Kazan, Russia; 8 Extreme Biology Laboratory, Institute of Fundamental Medicine and Biology, Kazan Federal University, 420009 Kazan, Russia; islamka32@gmail.com (I.N.); ruselusalbus@gmail.com (R.D.); rjuka@mail.ru (E.S.); 9 RIKEN Center for Integrative Medical Sciences, RIKEN, Yokohama, Kanagawa 230-0045, Japan; 10 Department of Functional Transcriptomics for Medical Genetic Diagnostics, Graduate School of Medicine, Juntendo University, Tokyo 113-8421, Japan

**Keywords:** skeletal muscles, promoters, transcriptomics, atrophy, enhancers, rat, cis-regulatory elements, RNA transcription, disuse, transcribed non-coding elements of genome

## Abstract

**Simple Summary:**

The genetic process underlying the control of skeletal muscle homeostasis is a key factor in methods that develop technologies to prevent age and immobility-driven atrophy. In the current paper, using advanced methods for the whole-genome profiling of transcription starting sites in fast and slow muscle in rats, we developed an integrative database of transcribed regulatory elements. Employing methods of comparative transcriptomics, we demonstrate that cis-regulatory elements are actively involved in the control of atrophy and recovery, and that the differential use of promoters and enhancers is the one of the key mechanisms that distinguishes between specific processes in slow and fast skeletal muscles.

**Abstract:**

The prevention of muscle atrophy carries with it clinical significance for the control of increased morbidity and mortality following physical inactivity. While major transcriptional events associated with muscle atrophy-recovery processes are the subject of active research on the gene level, the contribution of non-coding regulatory elements and alternative promoter usage is a major source for both the production of alternative protein products and new insights into the activity of transcription factors. We used the cap-analysis of gene expression (CAGE) to create a genome-wide atlas of promoter-level transcription in fast (m. EDL) and slow (m. soleus) muscles in rats that were subjected to hindlimb unloading and subsequent recovery. We found that the genetic regulation of the atrophy-recovery cycle in two types of muscle is mediated by different pathways, including a unique set of non-coding transcribed regulatory elements. We showed that the activation of “shadow” enhancers is tightly linked to specific stages of atrophy and recovery dynamics, with the largest number of specific regulatory elements being transcriptionally active in the muscles on the first day of recovery after a week of disuse. The developed comprehensive database of transcription of regulatory elements will further stimulate research on the gene regulation of muscle homeostasis in mammals.

## 1. Introduction

There are more than 600 different skeletal muscles in the human body, representing a complex and heterogeneous system capable of remodeling in response to mechanical load. The specific structures of fibers containing multiple nuclei make skeletal muscles very attractive, but a complicated case for studies aimed at explaining the contribution of gene expression to the maintenance of homeostasis. Long-term muscle unloading, which is observed in such conditions as bed rest, limb immobilization, or space flight, induces loss of muscle mass, strength, and function, accompanied by muscle fiber transition from a slow to fast phenotype [1] and leads to muscle atrophy [2]. On the other hand, activity and mechanical loading result in muscle hypertrophy and muscle growth and recovery. The prevention of muscle atrophy has clinical significance in the control of increased morbidity and mortality following physical inactivity and microgravity. On the molecular level, muscle atrophy is associated with large changes in the interplay between protein synthesis and degradation, with the involvement of the activity of a number of genes collectively referred to as ‘atrogenes’, including members of the ubiquitin–proteasome and the autophagy–lysosomal pathways [3]. While major transcriptional events associated with muscle atrophy–recovery processes are elucidated to some extent on a gene level, little is known about the involvement of non-coding regulatory elements. At the same time, the utilization of different promoters of the same gene is one of the major sources of both the production of alternative protein products and new deep insights into the activity of transcription factors [4]. Moreover, recent advances in transcriptomics reveal a key role of promoter- and enhancer-associated RNAs in the identification of cis-regulatory elements and the control of gene expression [5].

Thus, in the current study, using the cap analysis of gene expression (CAGE), we created a whole-genome single nucleotide-level atlas of the expression of transcription starting sites in the muscles of rats subjected to hindlimb unloading and subsequent recovery to discover novel, and still unidentified members of the atrophy–recovery process and to assess the contribution of regulatory elements to the rearrangement of muscle fibers. We found that both slow–fast fiber transition and atrophy–recovery processes in the muscles involve active differential promoter usage in the same genes.

## 2. Materials and Methods

### 2.1. Animals

Twenty-one mature Wistar rats, weighing 289 ± 57 g, were housed in a temperature- (20–21 °C), and humidity-controlled environment with a 12/12 light/dark cycle and access to food and water ad libitum. All procedures with animals were carried out in accordance with international bioethical standards [6], approved by the Commission on Biomedical Ethics of the State Research Center of the Russian Federation, Institute of Biomedical Problems of the Russian Academy of Science (protocol No. 319 of 4 April 2013), as well as the Guide for the Care and Use of Laboratory Animals recommendations.

### 2.2. Experimental Design

**Figure 1:**
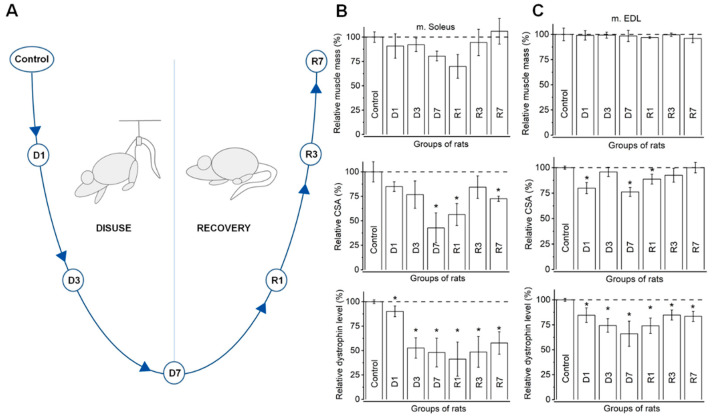
Design the U-experiment: morphometric and dystrophin fluorescence intensity analysis in muscle fibers during different time-points of disuse and recovery. (**A**) Time points of the muscle sampling during the disuse (Dx) and recovery (Rx) stage of the experiment. Estimation of relative muscle mass and relative cross-section area in m. Soleus (**B**) demonstrates significant decline after the seventh day of disuse to the first day of recovery, while in fast muscle, m. EDL (**C**) there are no significant changes. When assessing dystrophin fluorescence intensity in slow muscle m. Soleus (**B**), a significant decrease in dystrophin content during the third and seventh days of disuse, and the first day of the recovery period, is observed, followed by a slight increase until the seventh day of recovery. In m. EDL (**C**), a significant decline in dystrophin content after the third to the seventh day of disuse, and a rise on the first day of recovery, is also demonstrated. In total, 100% of the values for the control group were accepted. The significant differences are indicated by asterisks. See Supplementary Table ST1 for full data.

To study promoter- and enhancer-level regulation, and to identify patterns of differential gene expression in response to muscle disuse and recovery, we used the hindlimb unloading and reloading model that we called the U-experiment (Figure 1). To induce muscle atrophy, animals were unloaded in individual cages, hanging by their tails, as described by Morey-Holton and Globus [7]. Following 7-day disuse, a subset of rats was reloaded by releasing their tails from the wire and letting them actively move around the cage. In the study, two types of muscles were examined. “Fast” extensor digitorum longus (m. EDL) and “slow” m. soleus were collected from active rats (control group), after 1, 3, and 7 days of unloading and after 1, 3, and 7 days of reloading, following 7-day hindlimb unloading. The control and each time-point groups contained three individuals. Animals were euthanized by decapitation under deep ether anesthesia. One-side muscle samples were fast frozen in liquid nitrogen, and the others were weighed and fixed for immunochemistry.

### 2.3. Immunochemistry

Extirpated muscles were fixed in a 4% buffered paraformaldehyde solution (pH = 7.2) prepared with phosphate buffer (phosphate-buffered saline PBS; Sigma-Aldrich, St. Louis, MO, USA ). For cryoprotection, the fixed material was placed in a 30% sucrose solution (Sigma, USA), prepared in phosphate-buffered saline containing 0.1% sodium azide (Sigma, USA), for 24 h. Cross-sections (9 μm thick) were obtained on a HM 560 Cryo-Star cryostat (Carl Zeiss, Jena, Germany) using a water-soluble medium (Tissue-Tek^®^ O.C.T.™ Compound, Sakura Finetek, Torrance, CA, USA ). Series of muscle cross-sections were mounted on slides with a polylysine coating (ThermoFisher Scientific, Waltham, MA, USA ) and dried at room temperature for one hour. After washing three times for 5 min in a PBS solution containing a 0.5% solution of X−100 Triton (Sigma, USA), the samples were successively incubated for 30 min in solutions of 5% normal donkey serum and 1% bovine serum albumin. Then, the sections were incubated in a solution with primary antibodies to dystrophin for 12 h at 4 °C (1:250, rabbit, poly; 1:250, antibody Anti-Dystrophin ab15277 Rabbit polyclonal to Dystrophin Abcam Plc, Cambridge, United Kingdom). After that, the secondary antibodies (donkey IgG anti-rabbit) conjugated with Alexa488 fluorochrome (1:1000, Invitrogen, Waltham, MA, USA) for 1 h at room temperature. After staining, the sections were embedded in ImmuMount medium (ThermoFisher Scientific, Waltham, MA, USA).

Control experiments were performed to confirm the specificity of the binding of polyclonal antibodies to the corresponding proteins. For the negative control, the samples were incubated with secondary antibodies prior to incubation with primary antibodies. The absence of staining in the control experiments indicates the specificity of the antibodies binding to the corresponding peptides.

Images of micropreparations were obtained using a Leica TCS SP5 MP confocal scanning microscope, using an oil immersion lens 63 ×/ 1.4 and, a photomultiplier tube, using the LasX program (Leica, Wetzlar, Hessen, Germany). To stimulate the Alexa 488 fluorophore, argon and helium–neon lasers with wavelengths of 470–490 nm were used, and an emission filter with a passband of more than 515 nm was applied to detect luminescence. This work was carried out using the equipment of the Collective Spectroanalytical Center for Physical and Chemical Research of the Structure, Properties, and Composition of Substances and Materials of the Federal State Budgetary Institution of Science of the Federal Research Center of the Kazan Scientific Center of the Russian Academy of Sciences.

### 2.4. Morphometric and Statistical Analysis

All preparations were processed under the same conditions. The intensity of fluorescence and cross-sectional areas of muscle fibers were analyzed on obtained images using the ImageJ software ver.1.53c (NIH, Bethesda, MD, USA). For this purpose, three areas of 1024 × 1024 pixels in one slice were randomly selected, and 5 slices for each animal were examined. The immunofluorescence intensity in muscle fiber sarcolemma was estimated in Relative Fluorescent Units (RFU) after subtracting the background fluorescence according to software settings. Cross-sectional area (CSA) of muscle fibers was defined as the minimum internal diameter, since this parameter is least dependent on the muscle cutting angle [8] and is present in image processing programs.

The significance of differences between multiple groups was evaluated by one-way ANOVA. Muscle fiber size distributions were analyzed using Kruskal–Wallis non-parametric ANOVA with a significance level set at *p* < 0.05. To identify differences in muscle mass and dystrophin fluorescence intensity between groups, we used the nonparametric Mann—Whitney U-test. Data are presented as mean ± standard error. The control group was used for comparison with the experimental groups. To neutralize variations in physiological parameters that were related with differences in the body mass, all morphometric measures were normalized to animal weight. All of the mentioned statistical tests were performed using Origin 2019b software (OriginLab, Northampton, MA, USA).

### 2.5. RNA Isolation

Total RNA was isolated from frozen muscle tissues using RNeasy Fibrous Tissue Kit (Qiagen, Hilden, Germany), according to the manufacturer’s protocol. The concentration and purity of extracted RNA was measured by the NanoDrop™ 8000 Spectrophotometer (ThermoFisher Scientific, Waltham, MA, USA). The RNA quality was verified by Agilent Bioanalyzer 2100 (Agilent Technologies, Santa Clara, CA, USA ).

### 2.6. CAGE-Seq

Libraries were prepared according to the standard nAnT-iCAGE protocol (non-Amplified non-Tagging Illumina Cap Analysis of Gene Expression) [9]. In total, 2.5–3 μg total RNA was used as a template for the synthesis of the first cDNA strand (nAnT-iCAGE Library Preparation kit DNAform, Yokohama, Japan and SuperScript III Reverse Transcriptase, Invitrogen, Waltham, MA, USA), which was then biotinylated at the 5′- end using (nAnT-iCAGE Library Preparation kit, DNAform, Yokohama, Japan). This allowed for the selection of the 5′-cap containing RNAs using streptavidin beads (Dynabeads M-270 Streptavidin, ThermoFisher Scientific, USA). At this stage, rRNA, truncated or not fully transcribed RNA, was eliminated. For the more efficient removal of non-specific RNA strands, cDNA was treated by RNase I and H (nAnT-iCAGE Library Preparation kit, DNAform, Japan) and then purified using RNACleanUP (Beckman Coulter, Brea, CA, USA). Next, the linkers at the 5′and 3′ ends (nAnT-iCAGE Library Preparation kit, DNAform, Japan) were sequentially ligated to the cap-trapped cDNA. The 5′- linker contains the XmaJI restriction endonuclease recognition site (nAnT-iCAGE Library Preparation kit, DNAform, Japan) and the MmeI class II restriction enzyme recognition site (nAnT-iCAGE Library Preparation kit, DNAform, Japan), as well as a barcode for multiplexing. The 3′- linker contains an XbaI restriction enzyme recognition site (nAnT-iCAGE Library Preparation kit, DNAform, Japan). After treatment with these restriction enzymes, short CAGE tags were obtained to which a sequencing primer was ligated.

At the final stage, a second cDNA strand was synthesized (nAnT-iCAGE Library Preparation kit, DNAform, Japan). The concentration of the libraries was measured by PicoGreen Assay on a GloMax^®^ Multi Detection System (Promega, Madison, WI, USA), and the quality of the libraries was checked using an Agilent Bioanalyzer 2100 (Agilent Technologies, Santa Clara, CA, USA). Finally, the libraries were validated using real-time PCR (KAPA Library Quantification Kits Illumina, KAPA Biosystems, Wilmington, MA, South Africa) and sequenced on a HiSeq 2500 platform (Illumina, San Diego, CA, USA) using the HiSeq v4 reagent kit (HiSeq SR Cluster Kit v4 cBot and HiSeq SBS Kit v4 50 cycles, Illumina, San Diego, CA, USA) in the 50 bp single-end mode.

### 2.7. Data Analysis

Single-read sequences were analyzed for quality and over-represented adapter sequences with the FastQC tool. Quality filtering and adapter trimming were performed with the Trimmomatic tool v0.39. Read mapping on rat genome Rnor6 was performed with the STAR local alignment tool, version 2.6.1. CAGE tag start sites (CTSS) for each sample were imputed using the PromoterPipeline aggregation script from the C1 CAGE protocol. Peak clustering was performed with the decomposition-based Peak Identification (DPI1) tool. Bidirectional enhancers were identified using the pipeline by Andersson et al. 2014. The statistical significance of the differential expression of TSS and CTSS peaks was calculated using the DeSeq2 tool.

#### 2.7.1. Quality Control

For adapter clipping, we used a maximum mismatch count of 2, a palindrome threshold of 30, and a simple threshold of 10. For sliding window quality trimming, we used a window size of 4 nucleotides and the average PHRED score over the window was required to be above 15. The leading and trailing nucleotides of each read were removed if their quality was less than 3. All reads with length less than 36 were discarded.

#### 2.7.2. Genome, Annotation, and Indexing

As a reference genome, we used the Rattus norvegicus Rnor_6.0 (build NCBI:GCA_000001895.4) “top level” assembly acquired from Ensembl v93. As a source of annotated spice junctions for genome indexing, we used Ensembl v93 *R.* norvegicus genome annotation.

#### 2.7.3. Read Mapping

For local alignment of the reads onto the *Rattus norvegicus* genome Rnor_6.0 (build NCBI:GCA_000001895.4) “top level” assembly, we initially allowed for multimap reads to map up to 20 loci. We also required a minimum splice alignment overhang of 8 nt for de novo splice junctions (—alignSJoverhangMin 8) and 1 nt for splice junctions used from genome annotation (—alignSJDBoverhangMin 1). Spliced alignments were filtered using output splice junctions (—outFilterType BySJout), as the maximum number of mismatches per pair, relative to the read length default value of 0.04, was used (—outFilterMismatchNoverReadLmax 0.04) and the minimum intron length was set to 20 nt (—alignIntronMin 20) [10].

#### 2.7.4. CTSS Aggregation

All alignments were filtered for the absence of a “read unmapped” flag (flag value 4). Alignments that passed filtering were aggregated with the PromoterPipeline tool, version 2015.05.16, and the resulting CTSS were sorted by chromosome and coordinate.

#### 2.7.5. TSS Peaks

We clustered CTSS peaks with DPI1 in SPI mode in all samples of SOL and EDL muscles, respectively. Output peaks were filtered by maximum CTSS counts over the peak and maximum TPM normalized CTSS counts per peak. Thus, three sets of output peaks were obtained: unfiltered, ‘permissive’ (ctssMaxCounts3), and ‘robust’ (ctssMaxCounts11, ctssMaxTpm1).

#### 2.7.6. Enhancers

We estimated putative enhancers as loci expressing enhancer RNA (eRNA) from non-genic bidirectional promoters using the Enhancers software [11]. To avoid the false identification of intergenic enhancers, we masked all proximal loci, including known TSS (±500 nt) and exons (±200 nt), according to genome annotation. Enhancers were searched for among ‘permissive’ sets of TSS peaks of fast (EDL) and slow (soleus) muscle CTSS.

#### 2.7.7. Annotation of TSS Peaks to Genes and Enhancers

We annotated all TSS peaks to corresponding enhancers using the criteria of strandless coordinate intersection. To maintain consistency with enhancers, which are extended by 200 nt with the enhancer pipeline, we extended the gene regions by 200 nt from the 3′ and 5′ ends before intersecting them strand-wise with the permissive set of TSS peaks. Intersected peaks were annotated to corresponding genes.

#### 2.7.8. Differentially Expressed Peaks, Genes, and Enhancers

We used DeSEQ2 software to estimate the log2 fold change (LFC) of expression of each peak between the control and case samples, as well as the *p*-value as the probability of rejecting the null hypothesis, which stated that the mean expression of a peak is equal between the case and control samples. The *p*-values were adjusted using the Bonferroni–Hochberg false discovery rate method. The set of differentially expressed peaks for each case was identified by applying the cutoffs of |LFC| > 1.25 and Padj < 0.05. We considered an enhancer or gene differentially expressed if at least one of the peaks within the region of interest was identified as being differentially expressed.

#### 2.7.9. Gene Set Enrichment Analysis (GSEA)

We performed gene set enrichment analysis using the PANTHER Slim GO biological process database and webserver using the Fisher exact test and Bonferroni *p*-value adjustment. For observatory GSEA analysis, we also used the REVIGO web service with a permitted similarity of 0.5 (small set of results) against the *R. norvegicus* GO database.

## 3. Results and Discussion

### 3.1. Morphometric Analysis Confirms Distinct Disuse Associated Remodeling in Slow, but Not Fast Muscles

Skeletal muscle is a heterogeneous system characterized by high plasticity. Each muscle has a distinct composition of fiber types, determining its phenotype and its structural and functional properties. In mammalian skeletal muscle, four major (one slow (I) and three fast fiber types (IIa, IIb, and IIx)), and minor fiber types are classified (see [12]). Adaptation to various external stimuli (training or unloading) leads to muscle remodeling through the transition between fast and slow fibers.

It is known that slow-twitch muscles have decreased protein content when compared to fast-twitch muscle [13]. The soleus muscle is a postural slow-twitch muscle, which is preferentially affected by disuse, compared to other hindlimb muscles [14]. Extensor digitorum longus, being a fast-twitch muscle, is less vulnerable to muscle disuse than soleus [13]. The molecular–genetic mechanisms of muscle fiber shift in slow and fast muscle types at early stages of functional disuse, and readaptation to activity, still remains insufficiently understood.

In the study, we used hindlimb suspension as it is a commonly used rodent model in disuse and gravity unloading investigations [15]. Remarkably, these animals demonstrate no signs of chronic stress, which would provoke a change in their nutrition [14].

To confirm that disuse-mediated muscle atrophy occurred in suspended rats, we collected morphometric and biochemical data by estimating both EDL (fast muscle) and soleus muscle (slow muscle) mass, the cross-sectional area of muscle fibers, and dystrophin level in the control and each of the experimental groups using immunostaining. To minimize variations in the physiological parameters related to differences in the body mass, all physiological measures were normalized to body mass, then changes in physiological measures were expressed in percent relative to the control animals (Figure 1).

Muscle mass estimation demonstrated a reduction in slow muscle mass after seven days of disuse and in the first day of recovery (Figure 1B), followed by a sharp rise by the third and seventh days of recovery to values exceeding those in the control group. On the contrary, there were no significant changes in the masses of fast muscles throughout the experiment (Figure 1C).

The estimation of the cross-sectional area of muscle fibers demonstrated a trend in slow muscle in response to disuse and recovery for the muscle mass assessment. A significant decrease in fiber relative CSA from 7.2 ± 0.7 μm^2^/g in the control group to 3.0 ± 0.4 μm^2^/g on the seventh day of disuse was shown (Figure 1B). After the first day of recovery, relative CSA slightly increased to 4.0 ± 0.4 μm^2^/g (*p* ≤ 0.05) and reached 5.2 ± 0.1 μm^2^/g on the seventh day. In fast muscles, we observed a significant decline in relative CSA on the first (3.4 ± 0.1 μm^2^/g) and seventh day (3.2 ± 0.1μm^2^/g) of disuse, and a slight rise on the first day of recovery (3.8 ± 0.1 μm^2^/g) (Figure 1C).

Fluorescence intensity analysis also showed a significant reduction in relative dystrophin content in slow muscle during disuse (Figure 1B). The dystrophin fluorescence intensity vastly dropped to 118.4 ± 12.3 relative units (r.u.) on the third day of disuse compared with the control group (224.9 ± 4.2 r.u.) and to 107.9 ± 15.9 r.u. on the seventh day of disuse. A decline of up to 92.6 ± 16.1 r.u. continued on the first day of the recovery period. Then, we observed an increase in dystrophin intensity to 109.3 ± 17.2 (*p* ≤ 0.05) and 129.9 ± 15.0 (*p* ≤ 0.05) r.u. on the 3rd and 7th days of recovery, respectively; nevertheless the dystrophin values did not return to those of the control.

In contrast to the dynamics of muscle mass and CSA, fluorescence intensity analysis in fast muscle showed a dystrophin alteration pattern that was similar to that in slow muscle. There was a significant decrease in dystrophin fluorescence intensity from 244.2 ± 3.4 r.u. in the control group to 181.5 ± 12.2 (*p* ≤ 0.05) and 161.2 ± 20.4 (*p* ≤ 0.05) r.u. on the 3rd and 7th days of disuse, respectively. There was a minor significant increase to 180.8 ± 13.8 r.u., 207.0 ± 10.4 r.u., and 203.8 ± 10.4 r.u. after 1, 3, and 7 days of returning animals to normal physical activity, respectively (Figure 1C).

The above evidence confirms atrophic and readapted changes in both “slow” and “fast” muscle types in rats. Thus, the applied model is a representative selection for the construction of the atlas of genomic regulatory element activity during disuse and recovery.

### 3.2. Integrative Database of Rat’s Regulatory Elements with a Focus on Muscles

The TSS expression results of the current study were integrated with all available data of rat promoter-level expression data from various sources, including FANTOM5 (https://fantom.gsc.riken.jp/5/ (accessed 17 June 2021)). The integrated resource, reflecting the current state of knowledge, is now accessible as a special “Rat Regulome” sub-section of the GTRD database https://gtrd.biouml.org/ (accessed 17 June 2021) [16]. The incremented GTRD-based resource provides a web interface (Supplementary Materials “GTRD web interface for CAGE-seq data”) for visualization and expression analysis of the date with options to download the data of interest, as well as to view the tracks in the BioUML [17,18] and UCSC genome browsers.

We further developed a new set of MySQL tables that are used by the web interface for the GTRD database and convert data to bigWig and bigBed to visualize them in the BioUML or UCSC genome browsers. As there were no unified reference sets of TSS for rats, we took the initiative to establish a unified reference system for TSS, derived in the current projects, as well as for all available resources, including FANTOM5. For the incremental building of a robust set of TSSs, promoters, and enhancers with stable identifiers, we created a master track that contains all required information for further analysis. Master track is a bigBed file that contains a special JSON field that stores extensive information about corresponding sites. For example, TSS contains profiles for each group of experiments, promoters/enhancers associated with overlapping gene regions (TSS, exon, intron, 5′UTR, and 3′UTR), and nearest known TSSs (using different gene annotations). Master track includes both robust and permissive TSSs. These permissive TSSs can be further supported by other CAGE-seq experiments. In this case, information for permissive TSSs can be valuable for the more precise determination of TSSs boundaries. In brief, we have generally revealed more TSSs for each muscle type than in FANTOM5; however, they are related with smaller numbers of genes. Larger number of TSSs can be explained by deeper coverage in our experiment, compared to FANTOM5 data on rat tissues (see Supplementary Table ST2). A smaller number of genes can be explained as FANTOM5 covers more tissue types (aortic smooth muscle, hepatocytes, mesenchymal stem cells, universal RNA samples), while in our experiment we specifically investigated only skeletal muscles. However, due to deeper coverage and different physiological influences (disuse and recovery), we revealed many new TSSs both for the genes already described in FANTOM5 and those unique to the experiment. Further analysis of revealed TSSs allowed for grouping some of them into enhancers, so this experiment provides a new set of enhancers functioning in two types of skeletal muscles in different physiological conditions.

### 3.3. Muscle Atrophy and Recovery are Associated with Large Changes in the Unique Gene Set Expression Profiles, with Distinct Dynamics on Each Stage

**Figure 2:**
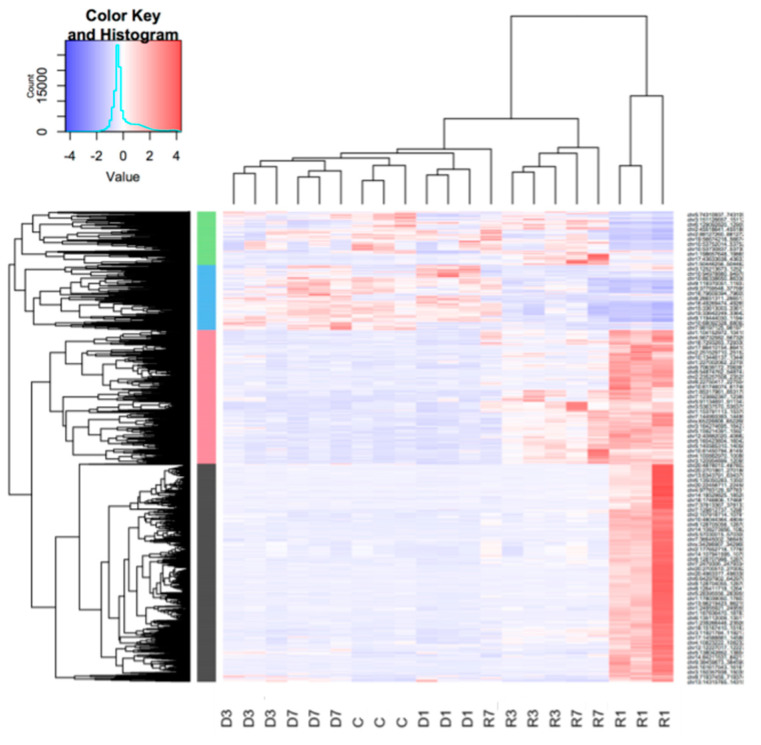
Clustering of differentially expressed peaks (both genic and intergenic), common to both phase-control and time-course comparisons (‘slow’ muscle—m. soleus). FDR threshold was 5 × 10^−4^. Clusters of the metabolism regulation through MAP kinase activity, actin biosynthesis, nucleosome assembly, and lipid metabolism are shown in black, green, blue, and red, respectively (see Supplementary Figure S1 for full figure). Sample names attributed to Dx—days of disuse, and Rx—recovery, and the numbers 1–3 at the end of the sample refer to the experimental animal.

To aggregate the CAGE tag start sites (CTSS) into peaks of TSS, we employed the DPI1 clustering tool. After applying a permissive threshold (see Methods section and Supplementary Data) there were 170,875 TSSs, associated with 13,337 genes in total in slow muscle, and 126,885 TSS associated with 11,915 genes in fast muscle (for details, see Supplementary Table ST3). Processes of atrophy and recovery in both muscles were associated with large changes in RNA transcription profile. The most dramatic changes in gene expression were observed in the slow muscles on the first day of recovery, both in phase vs. control (see Supplementary Table ST4A,B) and time-course (see Supplementary Table ST4C,D) comparisons. In contrast, the fast muscle showed comparatively weaker changes in expression during the whole recovery process. To note, the differentially expressed gene (DEG) composition signatures of the three different recovery phases of the fast muscle had only a small number of overlapping features. Both phase-control and time course signatures performed well as phase classifiers, except for one animal measured on the last day of recovery. The same applied to the statistics of the DEGs. Most of the peak signatures were common to both phase-control and time-course comparison, and the common set formed three clusters of expression across all experimental phases (Figure 2).

**Figure 3:**
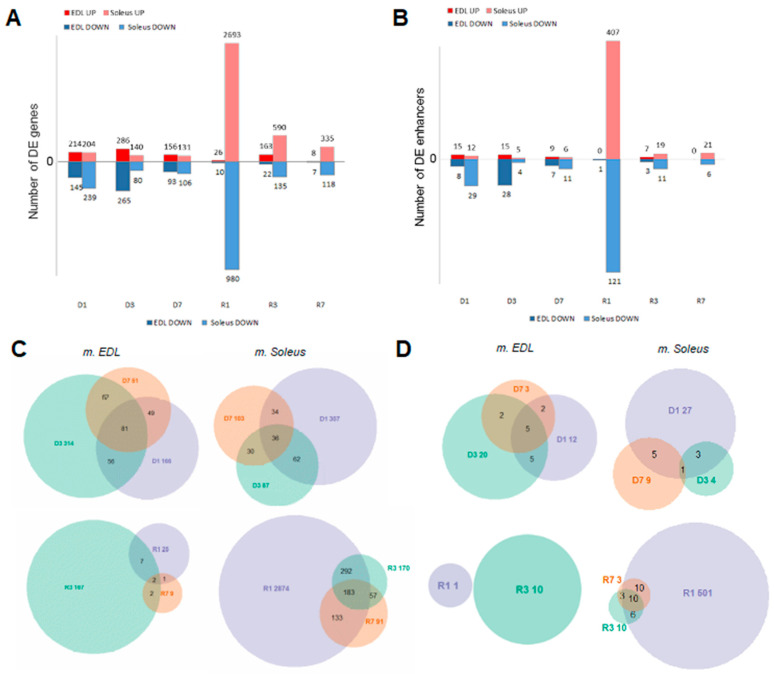
Differentially expressed genes (DEGs) and differentially expressed enhancers in soleus and EDL muscles. (**A**) Number of DEGs. (**B**) Number of differentially expressed enhancers. (**C**) DEGs in the fast and slow muscles (as compared to control) on days 1, 3 and 7 of disuse (shown as D1, D3, D7, respectively) and on days 1, 3, and 7 of recovery (shown as R1, R3, R7, respectively). (**D**) Differentially expressed enhancers in the fast and slow muscles (as compared to control) on days 1, 3 and 7 of disuse (shown as D1, D3, D7, respectively) and on days 1, 3, and 7 of recovery (shown as R1, R3, R7, respectively).

The differentially expressed genes in the slow muscle on the first day of recovery also outnumbered any other phase of the experiment several-fold. Interestingly, in the fast muscle, the number of DEGs on the first day of recovery was dramatically lower than during the other phases of the experiment, except for the seventh day of recovery (Figure 3A).

Remarkably, most DEGs, as in recovering fast muscles, were specific to a certain phase of recovery (Figure 3C), which was not the case in the disused fast muscles or both disused and recovering slow muscles. Such divergence suggests the phase specificity of pathways involved in the recovery of fast muscle. In contrast, most time-course DEGs in slow muscles on the third day of recovery, compared to the first day of recovery, were the same as on the first day of recovery, compared to the last day of disuse (see Supplementary Table ST5).

### 3.4. Gene Enrichment Analysis Reveals that Adaption to Disuse Processes in Slow and Fast Muscles is Mediated by Different Molecular Pathways

To dissect the genetic processes specific for each phase of the atrophy–recovery process, we conducted analogous Gene Set Enrichment Analysis (GSEA) for DEGs that are unique in each phase (D1-3-7 and R1-3-7). (Refer to DEGs on each phase and PANTHER results in SM.) In agreement with DEGs data in each phase, we observed significant differences in GSEs for unique differentially expressed genes between slow and fast muscles. The “regulation of adaptive immune response” and “related T cell immunity” were the only enriched terms in the last disuse phase (day 7 of unloading experiment) in slow muscle, while the regulation of adaptive immune response and ATP nucleotide metabolic processes were presented in the enrichment set at days 1 and 3 of disuse in slow muscle, correspondingly. GSEs of the unique DEGs for recovery were also distinct between slow and fast muscles. Thus, in R1, the unique signatures in fast muscle were enriched with cell proliferation terms (see Supplementary Table ST5), while the unique signatures in slow muscle were enriched with terms related to metabolism (see Supplementary Table ST6). Notably, gene set enrichments in the slow muscle are much more widely represented in each recovery phase and demonstrate consistent functional pattern during recovery in this type of skeletal muscle: involvement of signaling (via G-protein-coupled receptor and phosphorylation cascades of intermediate and target proteins) and metabolic processes (cellular respiration, nucleotides biosynthesis, actin filament organization) in the early phase R1, follow-up activation of translational complex machinery in R3, and cell development and differentiation in the R7 phase.

Unlike in fast muscle, differentially expressed genes in slow muscle, which were common to phase-control and time-course comparisons, formed four clusters, the two largest of which were enriched with features of metabolism regulation through MAP kinase activity and actin biosynthesis. The other two demonstrated nucleosome assembly coordinated with muscle contraction and metabolism (see Figure 2). According to the heatmap, MAP kinase regulation was activated only on the first day of recovery (R1 phase) and actin biosynthesis lasted for the whole recovery period, this being the most expressed in R1. Notably, members of the nucleosome assembly pathways were active only in disuse and metabolism features were downregulated in the initial stages of recovery (Figure 2). 

To take a wider look at the specific disuse-associated changes, we further performed a similar analysis for each phase of the experiment, as compared to the control animals (see Supplementary Table ST7). In the slow muscle, most of the enriched terms were related to parent classes of muscle tissue remodeling and metabolism. Expectantly, enriched features of actin cytoskeleton (sarcomere) reorganization were up-regulated in recovery phases (R1–R7), and down-regulated mostly in disuse phases (D1–D7). Notably, a vast number of sarcomere features were downregulated in the early stages of recovery [19]. Surprisingly, downregulated signatures of R1 and R3 phases were enriched in terms of muscle cell differentiation and energy derivation (excluding the receptor signaling pathways), which is likely to be interpreted as a signature of the strong impact of stress associated with atrophic process on cell homeostasis.

According to our GSEA (Supplementary Table ST7), atrophic changes in biological processes in soleus muscle, such as the downregulation of actin organization, muscle differentiation, and metabolism had the highest expression, in terms of gene set enrichment, on the first day of recovery and not immediately upon disuse, as well as the greatest loss in the relative muscle mass of m. soleus (Figure 1B). Such an effect was not the case for the fast muscle (EDL). Thus, we assume that the processes of muscle atrophy in slow muscle, but not in fast muscle, are delayed both on morphometric and molecular levels.

### 3.5. Tissue-Specific Intergenic TSS Show Different Patterns in Slow and Fast Muscles and Widely Co-Express with Coding Genes in Response to Disuse

After the identification of the TSS profile in both muscles, we found that a substantial number of the identified peaks do not intersect either with Ensembl 99 or with RefSeq 106, or the 6.0 transcriptome annotation or FANTOM and, thus, were specific for muscle tissue (including 2246 out of 4149 DEGs in the slow muscle) and have never been described before (see Supplementary Table ST8).

**Figure 4:**
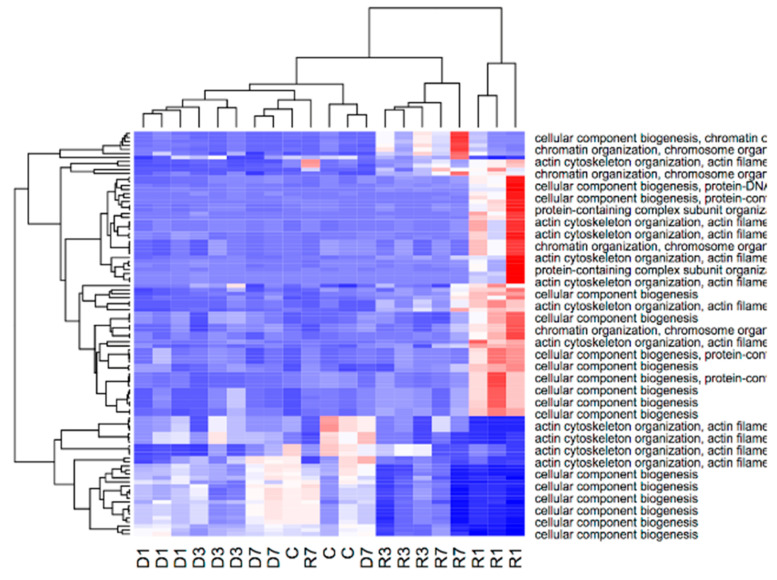
Non-genic peaks annotated with GO biological process of DEGs, which are located upstream (see Supplementary Figure S2 for full figure). Samples names attribute to Dx—days of disuse, and Rx—recovery.

A large number (2092) of intergenic TSSs also conformed to the aforementioned clusters along with the gene expression (Figure 3). Almost half of those intergenic TSSs (995) had a corresponding DEG as the closest downstream gene. Surprisingly, the inclusion of the differentially expressed intergenic TSS dramatically improved the transcriptome-based clustering of analyzed samples (see Figure 2 for clustering of genic peaks), which suggests strong, and yet to be explored in detail, involvement of alternative and distant promoters in the processes associated with muscle atrophy. These intergenic TSSs clustered along with robust genic signatures and also had a downstream DEG (394 genes in total) (Figure 3). As much as 152 of those DEGs were classified by GSEA analysis as signatures of chromatin reorganization, cytoskeleton organization, and cellular component biogenesis (see Supplementary Table ST9). The non-genic robust signature peaks upstream of those genes were also differentially expressed on the first day of recovery (see Figure 4), which is consistent with the GSEA analysis on DEGs.

**Figure 5:**
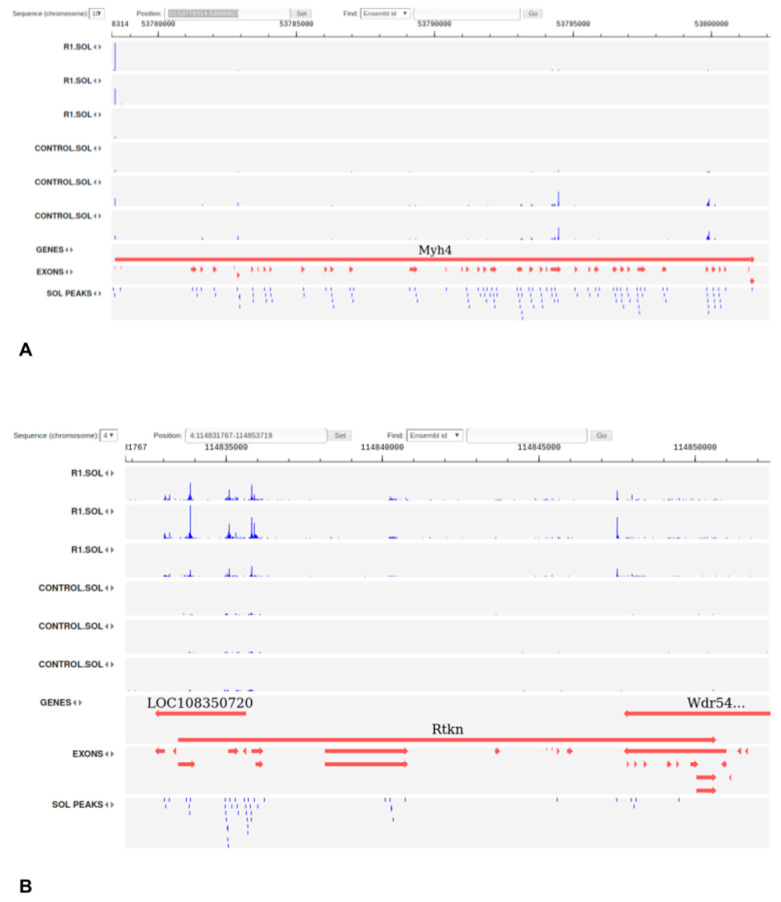
Alternative TSS in genes Myh4 (**A**) and Rtkn (**B**), shown in BioUML web browser. See Supplementary Figure S4 for the full figure.

In total, more than a half (1263 × 100/2246 = 56.23%) of the TSSs were associated with regions upstream of DEGs in slow muscle, and less than a half (210 × 100/474 = 44.30%) were region-associated TSSs in fast muscle, located upstream of a given DEG, which showed significant co-expression with that gene in the cycle of disuse—recovery (see Supplementary Table ST8, Figure 5B).

Taken together, we observed strong evidence of co-expression differentially using 5′UTRs and corresponding genes in the course of disuse-recovery, which illustrated the deep involvement of transcribed regulatory elements in the genetic control of muscle homeostasis (Figure 3A,B).

### 3.6. Alternative Usage of Dominating Transcription Starting Sites in Differentially Expressed Genes

We found that promoter switches play a substantial role in gene expression associated with atrophy processes in both types of muscles. For example, on the first day of recovery (R1 phase), the dominating (i.e., the most transcribed) TSS of the Myh4 gene was changed to the start of the full-length isoform, while the isoforms expressed in control samples were supposedly heavily 5′-truncated (Figure 5A).

In total, 24 genes, upregulated in slow muscle on the first day of recovery, had their dominating TSSs changed (see Supplementary Table ST10), including Dcn, which promotes muscle regeneration [20]; Ggnbp2, which regulates trophoblast proliferation [21]; Glul, which is involved in the regulation of glycogen synthesis via the up-regulation of glycogen synthase [22]; Limch1, which is unregulated as a result of endurance exercise in glycogen deficient mouse model [23]; and Pld3, which supposedly plays a role in myotube formation [24]. Interestingly, only 3 of those 24 genes were also upregulated and had their dominating TSSs changed on the first day of recovery in fast muscle–Bcurl, Syncrip, and Zmynd8.

Similarly, 28 genes were both upregulated and changed their dominating TSSs in fast muscle on the first day of recovery, including Ampd3, a subunit of AMP deaminase [25], Ankh, a mediator of cellular export of ATP; Dapk2, which is phosphorylated in a Ca^2+^-independent manner by AMPK in muscle tissue, undergoing dystrophy [26]; Flt1, a VEGF receptor, essential for skeletal muscle function [27]; Lpin1, which is essential for lipid metabolism and ATP synthesis [28]; Macf1, which maintains cellular components of microtubules in muscle [29]; Myl12a, myosin regulatory subunit; Prkag2, whose mutations are associated with skeletal muscle glycogenosis [30]; Xirp1, a marker of wounded skeletal muscle [31].

### 3.7. Expression of Transcribed Enhancers in Rat Skeletal Muscles is Linked to Disuse-Recovery Cycle

**Figure 6:**
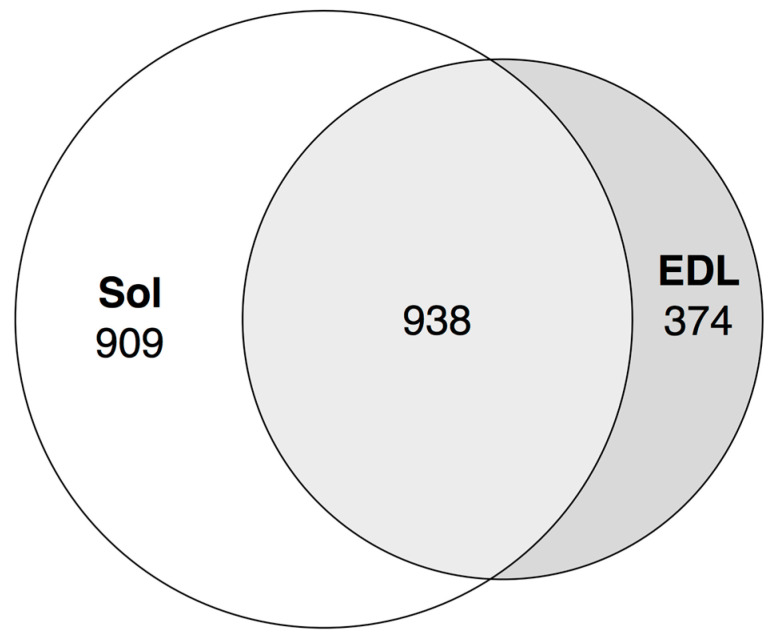
Tissue-specific enhancers of soleus and EDL muscles. Strandless coordinate intersection with 1 nt cutoff. See Supplementary Table ST11 for the full data.

We followed the enhancers pipeline [11] to determine bidirectionally expressed permissive DPI1 clusters, which were considered as enhancers. Less than 30% of fast muscle enhancers were distinct (did not intersect) from slow muscle enhancers, while roughly half of the soleus enhancers were distinct from fast muscle enhancers (Figure 6).

#### 3.7.1. Differentially Expressed Enhancers Mediate Muscle-Specific Transcription Program

In both slow and fast muscles, most of the enhancers were stably expressed in most of the phases of the experiment, except for R1, where the slow muscles exclusively made up approximately 30% of all detected enhancers that were differentially expressed (Figure 3B). Such a differential expression profile is consistent with that for differentially expressed intergenic promoters and genes (Figure 3A).

#### 3.7.2. Switch ON/OFF Enhancers on First Day of Recovery (R1)

We found that at least 26 enhancers in slow muscle were transcribed only on the first day of recovery (phase R1, see Supplementary Table ST12). These 26 enhancers had nine differentially expressed genes (AABR07004130.1, Stac2, Mn1, LOC102546495, Tsc22d2, AABR07044837.2, Pkdcc, Wnt1 and Ubash3b) in the reach of 1 Mb, all of which appeared upregulated (see Supplementary Table ST13).

In skeletal muscle, Stac proteins are essential for coupling membrane depolarization to Ca^2+^ release from the sarcoplasmic reticulum (SR). It was demonstrated that Stac proteins (1–3) interact with the II–III loop of CaV1.1, the principle subunit of the Ca^2+^ channel, mediating the excitation–contraction in skeletal muscles [32].

In the meantime, Wnt proteins are secreted ligands, which bind to distinct receptors, and the activation of the Wnt-mediated signaling pathway leads to GSK3 inhibition which, in turn, results in muscle hypertrophy. Moreover, in vitro treatment of the myotubes with Wnt induces cell differentiation, assuming an alternative induction for the phenotype [33].

In the Pkdcc gene encoding protein kinase domain-containing protein, cytoplasm has a broader functional role in organogenesis via the phosphorylation of extracellular proteins and endogenous proteins in the secretory pathway. It has been shown in only in several genome wide studies that Pkdcc is significantly associated with skeletal system development [33,34].

Remarkably, data analysis has identified Mn1, which encodes a transcriptional activator and plays a physiological role in cancer as an up-regulated gene during recovery, while the gene transcript was in a top-list of genes downregulated in the skeletal muscle after both neuromuscular electrical stimulation and resistance exercise [35]. Moreover, the recently published investigation of its physiological role has demonstrated that Mn1 is expressed to a greater extent in fetal and adult skeletal muscle compared to other human organs and tissues, and participates in the regulation of target genes through interaction with the transcription factors Pbx1, Pknox1, and Zbtb24 [36]. Taken together, the observed up-regulation of Mn1 expression in recovery stages can be explained by its pivotal role in developmental processes and cell differentiation [37,38].

The identification of Ubash3b upregulation, associated with corresponding enhancer switches, also represents biologically meaningful outcomes, since the gene encodes a protein that was found to inhibit the endocytosis of the epidermal growth factor receptor (Egfr) [39]. Endocytosis is the general path for cellular receptors such as Egfr to reduce the signaling response via negative feedback, whereas the Egfr-dependent signaling pathway promotes the functional rescue of dystrophin-deficient satellite cells and enhances skeletal muscle regeneration and strength in mice [40].

## 4. Conclusions

We conducted the first complex analysis of the landscape of transcription starting sites across different stages of disuse and recovery in two types of skeletal muscle, and showed that slow and fast muscles have different regulome, mediated by distinct sets of transcribed regulatory elements. Our study revealed that “shadow” enhancers, i.e., several enhancers that control the same target gene [41], are integral to the regulation of disuse-mediated processes in skeletal muscle. Moreover, the transcription activity of hundreds of enhancers among those identified in our study is strongly associated with specific stages of both atrophy and recovery processes in the muscles. The same is true for the RNA isoforms based on the differential use of transcription starting sites—there is a distinct set of transcripts (including at least several genes involved in maintaining muscles homeostasis) generated by employing the alternative promoters that are associated with different stages of both slow and fast muscle atrophy and recovery. We are confident that the atlas of regulatory element transcriptional activity, which we report in this paper, will serve as a platform for further studies of molecular regulatory mechanisms, controlling the recovery of muscles after atrophic processes and for expanding the medical genetic applications to non-coding regulatory elements in the rat genome.

## Data Availability

The atlas is available online as part of the GTRD database (http://gtrd.biouml.org/, sections *TSS**→Rattus norvegicus* and *Enhancers*→*Rattus norvegicus*). Transcriptome data are available at: https://www.ncbi.nlm.nih.gov/geo/query/acc.cgi?acc=GSE141827 (accessed on 17 June 2021).

## Supplementary Materials

The following are available online at https://www.mdpi.com/article/10.3390/biology10060557/s1. Figure SF1: Clustering of differentially expressed peaks, Figure SF2: Non-genic peaks annotated with GO biological process of upstream DEGs, Figure SF3: Morphometric analysis, Figure SF4: Alternative TSS in genes Myh4 and Rtkn, Table ST1: Morphometric analysis, Table ST2: Sample coverage, Table ST3: TSS Stats, Table ST4: Differentially expressed peaks, Table ST5: PANTHER enrichment in fast muscle (R1), Table ST6: PANTHER enrichment in slow muscle (R1), Table ST7: Fast and slow muscle – Gene Ontology, Table ST8: Muscle specific peaks and their downstream differentially expressed genes in muscles upon atrophy-recovery cycle, Table ST9: Phase-control and time-course clusters, Table ST10: Leading TSS in R1, Table ST11: Tissue-specific enhancers of slow and fast muscles, Table ST12: Swtch-on and switch-off enhancers, Table ST13: DEGs closest to the switch-on and switch-off enhancers, Supplementary Description (DOCX document).

## Abbreviations

CAGE	Cap Analysis Gene Expression
CSA	Cross-Sectional Area
CTSS	CAGE Tag Start Sites
DPI1	Decomposition-Based Peak Identification
EDL	Extensor Digitorum Longum
eRNA	enhancer RNA
GSEA	Gene Set Enrichment Analysis
LFC	Log2 Fold Change
RFU	Relative Fluorescent Units
TSS	Transcription Start Sites

## References

[1] Wang Y., Pessin J.E. (2013). Mechanisms for fiber-type specificity of skeletal muscle atrophy. Curr. Opin. Clin. Nutr. Metab. Care.

[2] Bodine S.C. (2013). Disuse-induced muscle wasting. Int. J. Biochem. Cell Biol..

[3] Ninfali C., Siles L., Darling D.S., Postigo A. (2018). Regulation of muscle atrophy-related genes by the opposing transcriptional activities of ZEB1/CtBP and FOXO3. Nucleic Acids Res..

[4] Piekarowicz K., Bertrand A.T., Azibani F., Beuvin M., Julien L., Machowska M., Bonne G., Rzepecki R. (2019). A muscle hybrid promoter as a novel tool for gene therapy. Mol. Ther. Methods Clin. Dev..

[5] Arnold P.R., Wells A.D., Li X.C. (2019). Diversity and emerging roles of enhancer RNA in regulation of gene expression and cell fate. Front. Cell Dev. Biol..

[6] Genin A.M., Il’in A.E., Kaplanskiĭ A.S., Kasatkina T.B., Kuznetsova K.A., Pestov I.D., Smirnova T.A. (2001). Bioethics of research on humans and animals in aviation, space and marine medicine. Aviakosm. Ekolog. Med..

[7] Morey-Holton E.R., Globus R.K. (2002). Hindlimb unloading rodent model: Technical aspects. J. Appl. Physiol..

[8] Briguet A., Courdier-Fruh I., Foster M., Meier T., Magyar J.P. (2004). Histological parameters for the quantitative assessment of muscular dystrophy in the mdx-mouse. Neuromuscul. Disord..

[9] Murata M., Nishiyori-Sueki H., Kojima-Ishiyama M., Carninci P., Hayashizaki Y., Itoh M. (2014). Detecting Expressed Genes Using CAGE. Methods Mol. Biol..

[10] Kouno T., Moody J., Kwon A.T.-J., Shibayama Y., Kato S., Huang Y., Böttcher M., Motakis E., Mendez M., Severin J. (2019). C1 CAGE detects transcription start sites and enhancer activity at single-cell resolution. Nat. Commun..

[11] Andersson R., Gebhard C., Miguel-Escalada I., Hoof I., Bornholdt J., Boyd M., Chen Y., Zhao X., Schmidl C., Suzuki T. (2014). An atlas of active enhancers across human cell types and tissues. Nature.

[12] Schiaffino S., Reggiani C. (2011). Fiber types in mammalian skeletal muscles. Physiol. Rev..

[13] Ohira Y., Yoshinaga T., Nomura T., Kawano F., Ishihara A., Nonaka I., Roy R.R., Edgerton V.R. (2002). Gravitational unloading effects on muscle fiber size, phenotype and myonuclear number. Adv. Space Res..

[14] Thomason D.B., Booth F.W. (1990). Atrophy of the soleus muscle by hindlimb unweighting. J. Appl. Physiol..

[15] Globus R.K., Morey-Holton E. (2016). Hindlimb unloading: Rodent analog for microgravity. J. Appl. Physiol..

[16] Kolmykov S., Yevshin I., Kulyashov M., Sharipov R., Kondrakhin Y., Makeev V.J., Kulakovskiy I.V., Kel A., Kolpakov F. (2021). GTRD: An integrated view of transcription regulation. Nucleic Acids Res..

[17] Valeev T., Yevshin I., Kolpakov F. (2013). BioUML genome browser. Virtual Biology.

[18] Kolpakov F., Akberdin I., Kashapov T., Kiselev L., Kolmykov S., Kondrakhin Y., Kutumova E., Mandrik N., Pintus S., Ryabova A. (2019). BioUML: An integrated environment for systems biology and collaborative analysis of biomedical data. Nucleic Acids Res..

[19] Williams G.P., Marmion D.J., Schonhoff A.M., Jurkuvenaite A., Won W.-J., Standaert D.G., Kordower J.H., Harms A.S. (2020). T cell infiltration in both human multiple system atrophy and a novel mouse model of the disease. Acta Neuropathol..

[20] Li Y., Li J., Zhu J., Sun B., Branca M., Tang Y., Foster W., Xiao X., Huard J. (2007). Decorin gene transfer promotes muscle cell differentiation and muscle regeneration. Mol. Ther..

[21] Li S., Moore A.K., Zhu J., Li X., Zhou H., Lin J., He Y., Xing F., Pan Y., Bohler H.C. (2016). Ggnbp2 is essential for pregnancy success via regulation of mouse trophoblast stem cell proliferation and differentiation. Biol. Reprod..

[22] Lin I.-H., Chang J.-L., Hua K., Huang W.-C., Hsu M.-T., Chen Y.-F. (2018). Skeletal muscle in aged mice reveals extensive transformation of muscle gene expression. BMC Genet..

[23] Fiuza-Luces C., Santos-Lozano A., Llavero F., Campo R., Nogales-Gadea G., Díez-Bermejo J., Baladrón C., González-Murillo Á., Arenas J., Martín M.A. (2018). Muscle molecular adaptations to endurance exercise training are conditioned by glycogen availability: A proteomics-based analysis in the McArdle mouse model. J. Physiol..

[24] Osisami M., Ali W., Frohman M.A. (2012). A role for phospholipase D3 in myotube formation. PLoS ONE.

[25] Fortuin F.D., Morisaki T., Holmes E.W. (1996). Subunit composition of AMPD varies in response to changes in AMPD1 and AMPD3 gene expression in skeletal muscle. Proc. Assoc. Am. Physicians.

[26] Shiloh R., Gilad Y., Ber Y., Eisenstein M., Aweida D., Bialik S., Cohen S., Kimchi A. (2018). Non-canonical activation of DAPK2 by AMPK constitutes a new pathway linking metabolic stress to autophagy. Nat. Commun..

[27] Verma M., Shimizu-Motohashi Y., Asakura Y., Ennen J.P., Bosco J., Zhou Z., Fong G.-H., Josiah S., Keefe D., Asakura A. (2019). Inhibition of FLT1 ameliorates muscular dystrophy phenotype by increased vasculature in a mouse model of Duchenne muscular dystrophy. PLoS Genet..

[28] Sellers R.S., Mahmood S.R., Perumal G.S., Macaluso F.P., Kurland I.J. (2019). Phenotypic Modulation of Skeletal Muscle Fibers in LPIN1-Deficient Lipodystrophic (fld) Mice. Vet. Pathol..

[29] Ghasemizadeh A., Christin E., Guiraud A., Couturier N., Risson V., Girard E., Soler C., Laddada L., Jagla K., Sanchez C. (2019). Muscle MACF1 maintains myonuclei and mitochondria localization through microtubules to control muscle functionalities. BioRxiv.

[30] Laforêt P., Richard P., Said M.A., Romero N.B., Lacene E., Leroy J.-P., Baussan C., Hogrel J.-Y., Lavergne T., Wahbi K. (2006). A new mutation in PRKAG2 gene causing hypertrophic cardiomyopathy with conduction system disease and muscular glycogenosis. Neuromuscul. Disord..

[31] Otten C., van der Ven P.F., Lewrenz I., Paul S., Steinhagen A., Busch-Nentwich E., Eichhorst J., Wiesner B., Stemple D., Strähle U. (2012). Xirp proteins mark injured skeletal muscle in zebrafish. PLoS ONE.

[32] Nelson B.R., Wu F., Liu Y., Anderson D.M., McAnally J., Lin W., Cannon S.C., Bassel-Duby R., Olson E.N. (2013). Skeletal muscle-specific T-tubule protein STAC3 mediates voltage-induced Ca^2+^ release and contractility. Proc. Natl Acad Sci USA.

[33] Glass D.J. (2005). Skeletal muscle hypertrophy and atrophy signaling pathways. Int. J. Biochem. Cell Biol..

[34] Liu Y., Shen H., Greenbaum J., Liu A., Su K.-J., Zhang L.-S., Zhang L., Tian Q., Hu H.-G., He J.-S. (2020). Gene Expression and RNA Splicing Imputation Identifies Novel Candidate Genes Associated with Osteoporosis. J. Clin. Endocrinol. Metab..

[35] Latimer L.E., Constantin D., Greening N.J., Calvert L., Menon M.K., Steiner M.C., Greenhaff P.L. (2019). Impact of transcutaneous neuromuscular electrical stimulation or resistance exercise on skeletal muscle mRNA expression in COPD. Int. J. Chron. Obstruct. Pulmon. Dis..

[36] Miyake N., Takahashi H., Nakamura K., Isidor B., Hiraki Y., Koshimizu E., Shiina M., Sasaki K., Suzuki H., Abe R. (2020). Gain-of-Function MN1 Truncation Variants Cause a Recognizable Syndrome with Craniofacial and Brain Abnormalities. Am. J. Hum. Genet..

[37] Meester-Smoor M.A., Vermeij M., van Helmond M.J.L., Molijn A.C., van Wely K.H.M., Hekman A.C.P., Vermey-Keers C., Riegman P.H.J., Zwarthoff E.C. (2005). Targeted disruption of the Mn1 oncogene results in severe defects in development of membranous bones of the cranial skeleton. Mol. Cell. Biol..

[38] Zhang X., Dowd D.R., Moore M.C., Kranenburg T.A., Meester-Smoor M.A., Zwarthoff E.C., MacDonald P.N. (2009). Meningioma 1 is required for appropriate osteoblast proliferation, motility, differentiation, and function. J. Biol. Chem..

[39] Kowanetz K., Crosetto N., Haglund K., Schmidt M., Heldin C.-H., Dikic I. (2004). Suppressors of T-cell receptor signaling Sts-1 and Sts-2 bind to Cbl and inhibit endocytosis of receptor tyrosine kinases. J. Biol. Chem..

[40] Wang Y.X., Feige P., Brun C.E., Hekmatnejad B., Dumont N.A., Renaud J.-M., Faulkes S., Guindon D.E., Rudnicki M.A. (2019). EGFR-Aurka Signaling Rescues Polarity and Regeneration Defects in Dystrophin-Deficient Muscle Stem Cells by Increasing Asymmetric Divisions. Cell Stem Cell.

[41] Waymack R., Fletcher A., Enciso G., Wunderlich Z. (2020). Shadow enhancers can suppress input transcription factor noise through distinct regulatory logic. eLife.

