# Supplementary material for: Genome-Wide Atlas of Promoter Expression Reveals Contribution of Transcribed Regulatory Elements to Genetic Control of Disuse-Mediated Atrophy of Skeletal Muscle
Source: Biology (Basel). 2021 Jun 20;10(6):557. doi: 10.3390/biology10060557 (PMC8235325; doi:10.3390/biology10060557)
Supplement: Supplementary file 1 [file biology-10-00557-s001.zip › biology-1143072-supplementary-reply_2.pdf]

Supplementary description

Morphometric analysis

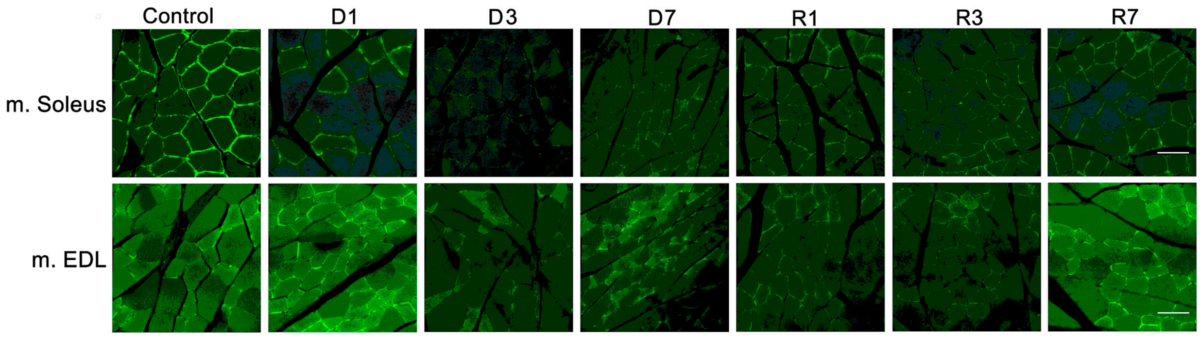

(See SF3 for full figure)

Images of m. Soleus and m. EDL cross-sections from control and experimental groups, stained by primary antibodies to dystrophin and secondary antibodies (donkey IgG anti-rabbit) conjugated to Alexa488 fluorochrome and obtained using a Leica TCS SP5 MP confocal scanning microscope. Scale: 50 µm.

Gene expression shifts associated with atrophy

All identified DPI1 peaks covered 17507 genes in EDL and 18599 genes. Permissive threshold (maximum CTSS count at least 3) decreased the gene coverage down to about 40% and the robust threshold (maximum CTSS count at least 11 and TPM normalization score at least 1) left roughly

a quarter of those genes covered. In our work we mostly relied on permissive threshold filtered peaks as TSS.

Threshold	soleus muscle		EDL muscle	
	peaks identified	genes covered	peaks identified	genes covered
No threshold	596908	18559	470041	17507
Robust	40632	7740	34196	6930
Permissive	170875	13337	126855	11915

Table SD1. DPI peaks identified in CTSS of EDL and SOL muscle cell

Comparison with FANTOM5

We have compared revealed TSSs in the above 2 muscle types with TSSs in the slow (soleus) and fast (EDL) rat muscle tissues described in FANTOM5. The results are shown in table SD2.

Generally, we have revealed more TSSs for each muscle type than in FANTOM5, however they are related with smaller numbers of genes. Bigger number of TSSs can be explained by deeper coverage in our experiment compared to FANTOM5 data on rat tissues (See ST2). Smaller number of genes can be explained that FANTOM 5 covers more tissue types (aortic smooth muscle, hepatocytes, mesenchymal stem cells, universal RNA samples) while in our experiment we have

specifically investigated only skeletal muscles. However due to deeper coverage and different physiological influences (disuse atrophy and recovery) we have revealed a lot of new TSSs both for genes already described in FANTOM5 and unique for the experiment.

Further analysis of revealed TSSs allowed us to group some of them into enhancers, so this experiment provides a new set of enhancers functioning in 2 types of skeletal muscles in different physiological conditions.

Thus results of the experiment significantly extend our knowledge about TSSs and enhancers functioning in rat skeletal muscles in different physiological conditions. To make it publicly available we put them into the GTRD database as described below.

	FANTOM5	Revealed in experiment			
		Total	Common with Fantom5	Unique	Muscle type
TSS	28 497	40 632<br>34 196	15 174<br>13 705	25 458<br>20 491	Soleus<br>EDL
Enhancers	NA	1 846<br>1 312		1 846<br>1 312	Soleus<br>EDL
Promoters					Soleus<br>EDL
Genes	8 351*	7 796<br>6 987	5 708<br>5 305	2 088<br>1 682	Soleus<br>EDL

Table SD2. Comparison of revealed rat TSSs with FANTOM 5

*Data obtained by intersecting FANTOM5 peaks with gene coordinates from Ensembl v99

Integration with FANTOM5 data

From a user view point it will be more convenient when obtained data will be integrated with FANTOM5 data. Main ideas are following:

- to build merged set of TSSs from FANTOM5 and obtained data;
- when TSSs are overlapping in FANTOM5 and obtained data, then maximally preserve FANTOM5 TSSs and in rare cases add new TSSs. This is the most sophisticated part. See its description below.
- using the joined set of TSSs to build the joined table of TSSs expressions using FANTOM5 and new data. For this purpose we are recalculating TSSs expressions using their joined set. Due to the suggested approach all FANTOM5 data will be preserved and seamlessly extended by the new data.
- annotate new TSSs using the same approach as FANTOM5. All FANTOM5 data also will be preserved and seamlessly extended by the new data.
- while FANTOM5 does not provide enhancers and their annotation for CAGE-seq data for rat, we are building a set of enhancers using both FANTOM5 and our data.
- to provide a unique ID for each TSS, enhancer and promoter so a user can unambiguously refer to them.

The suggested approach is used for incremental integration of other CAGE-seq data with FANTOM5 data. It was implemented as a new pipeline for the GTRD database that provides uniform annotation and analysis of wide range of NGS data related to gene expression regulation (Kolmykov et al., 2021; doi: 10.1093/nar/gkaa1057)

GTRD web interface for CAGE-seq data

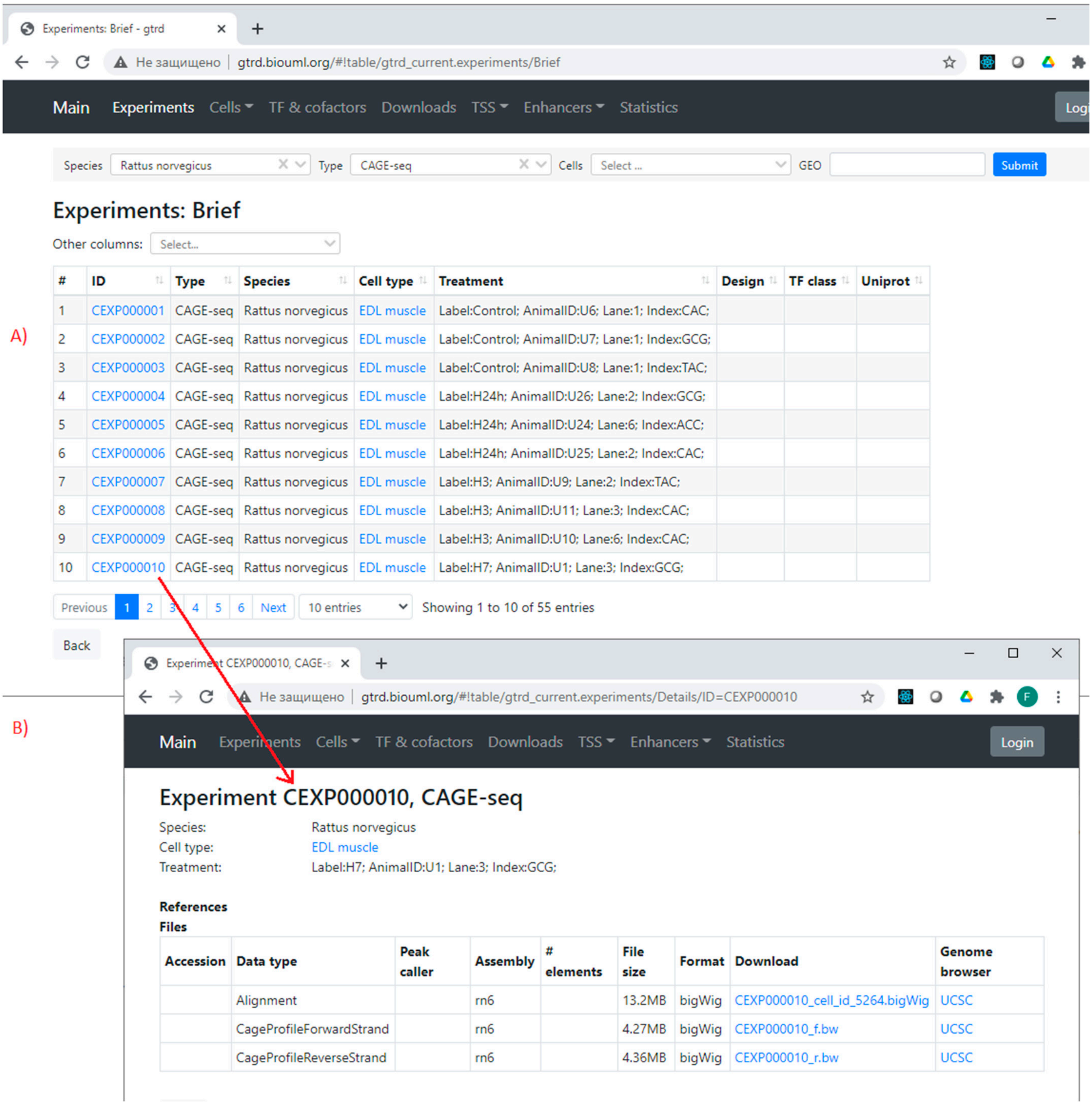

Figure SD1.List of CAGE-seq experiments (A) and detailed description of selected CAGE-seq experiment (B).

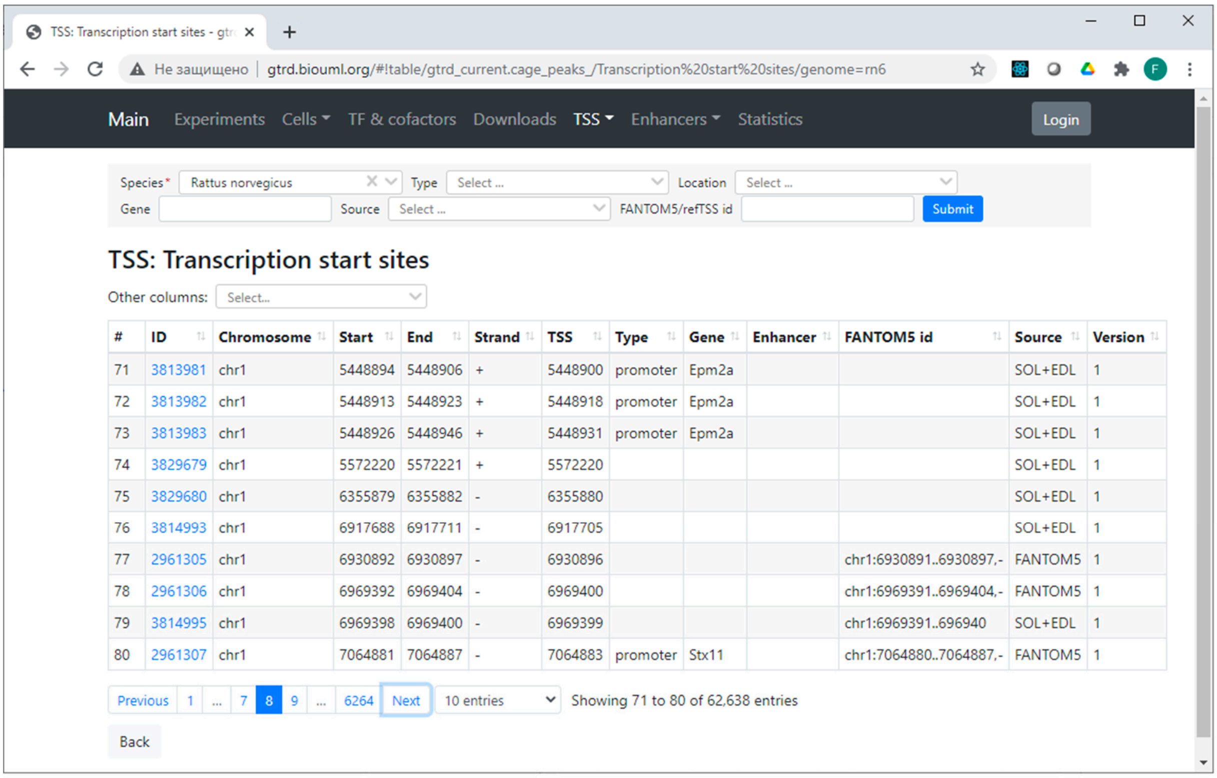

TSS: Transcription start sites - gtr

Не защищено | gtrd.biouml.org/#!table/gtrd_current.cage_peaks_/Transcription%20start%20sites/genome=rn6

Main Experiments Cells TF & cofactors Downloads TSS Enhancers Statistics Login

Species* Rattus norvegicus Type Select ... Location Select ...

Gene Source Select ... FANTOM5/refTSS id Submit

TSS: Transcription start sites

Other columns: Select...

#	ID	Chromosome	Start	End	Strand	TSS	Type	Gene	Enhancer	FANTOM5 id	Source	Version
71	3813981	chr1	5448894	5448906	+	5448900	promoter	Epm2a			SOL+EDL	1
72	3813982	chr1	5448913	5448923	+	5448918	promoter	Epm2a			SOL+EDL	1
73	3813983	chr1	5448926	5448946	+	5448931	promoter	Epm2a			SOL+EDL	1
74	3829679	chr1	5572220	5572221	+	5572220					SOL+EDL	1
75	3829680	chr1	6355879	6355882	-	6355880					SOL+EDL	1
76	3814993	chr1	6917688	6917711	-	6917705					SOL+EDL	1
77	2961305	chr1	6930892	6930897	-	6930896				chr1:6930891..6930897,-	FANTOM5	1
78	2961306	chr1	6969392	6969404	-	6969400				chr1:6969391..6969404,-	FANTOM5	1
79	3814995	chr1	6969398	6969400	-	6969399				chr1:6969391..696940	SOL+EDL	1
80	2961307	chr1	7064881	7064887	-	7064883	promoter	Stx11		chr1:7064880..7064887,-	FANTOM5	1

Previous 1 ... 7 8 9 ... 6264 Next 10 entries Showing 71 to 80 of 62,638 entries

Back

Figure SD2. Joined list of rat TSS in GTRD database.

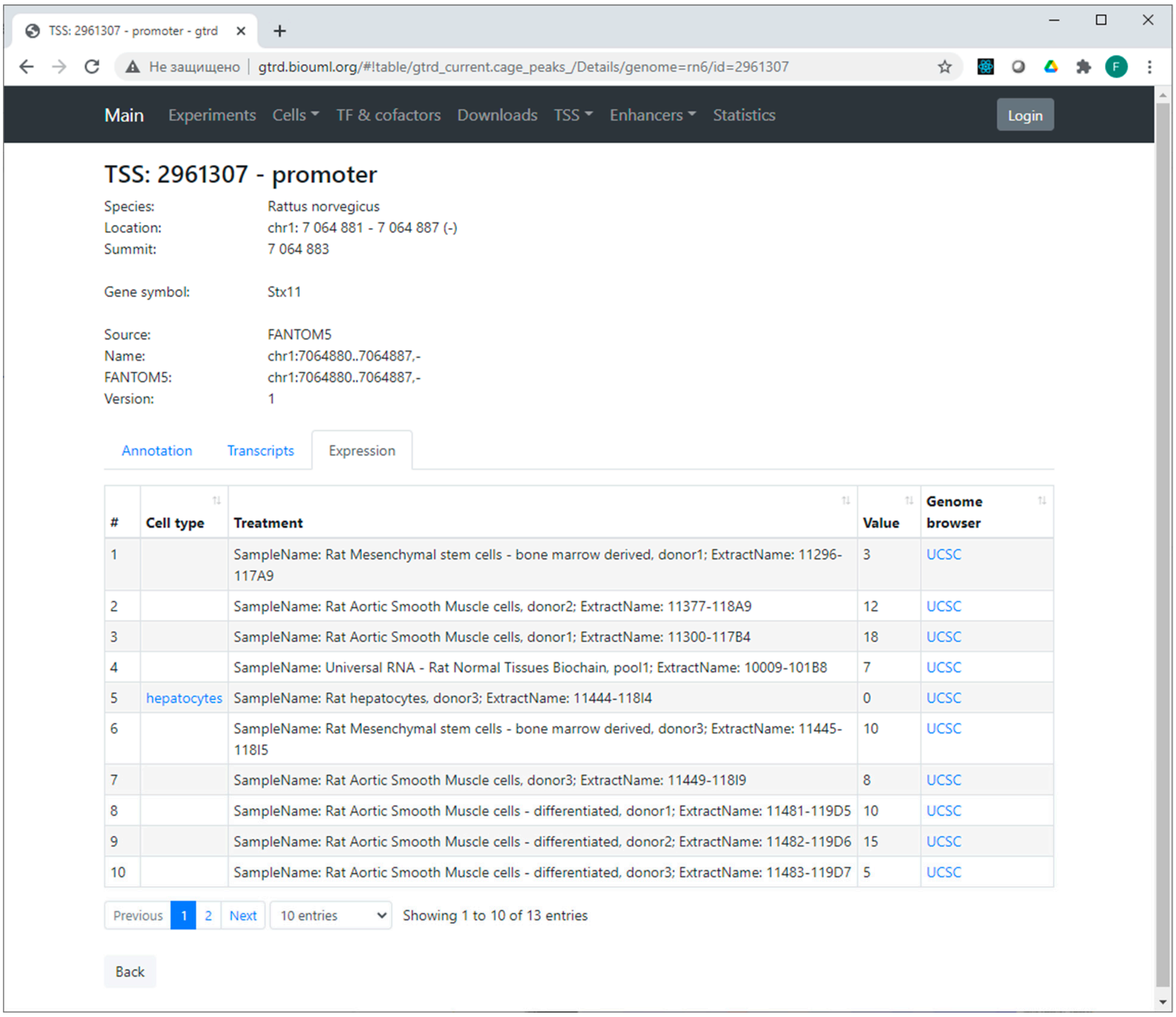

Figure SD3. Detailed description of selected rat promoter in GTRD database.

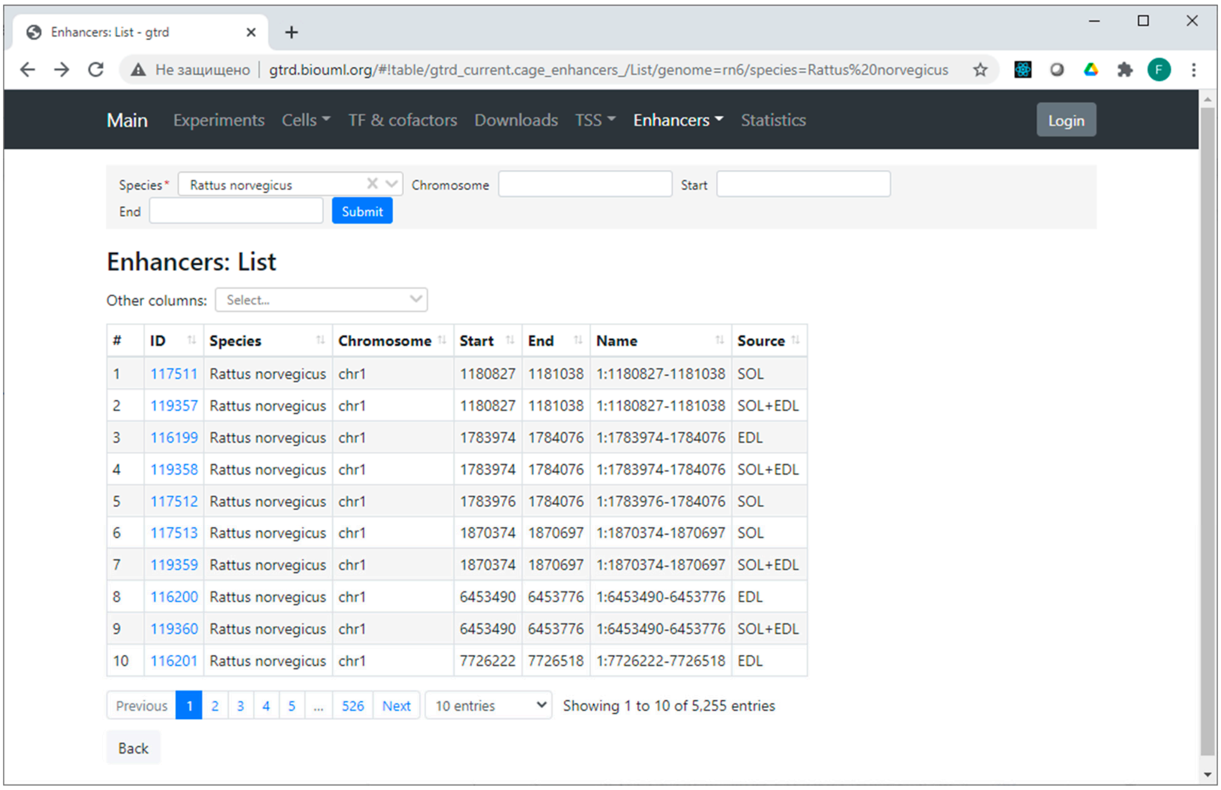

#	ID	Species	Chromosome	Start	End	Name	Source
1	117511	Rattus norvegicus	chr1	1180827	1181038	1:1180827-1181038	SOL
2	119357	Rattus norvegicus	chr1	1180827	1181038	1:1180827-1181038	SOL+EDL
3	116199	Rattus norvegicus	chr1	1783974	1784076	1:1783974-1784076	EDL
4	119358	Rattus norvegicus	chr1	1783974	1784076	1:1783974-1784076	SOL+EDL
5	117512	Rattus norvegicus	chr1	1783976	1784076	1:1783976-1784076	SOL
6	117513	Rattus norvegicus	chr1	1870374	1870697	1:1870374-1870697	SOL
7	119359	Rattus norvegicus	chr1	1870374	1870697	1:1870374-1870697	SOL+EDL
8	116200	Rattus norvegicus	chr1	6453490	6453776	1:6453490-6453776	EDL
9	119360	Rattus norvegicus	chr1	6453490	6453776	1:6453490-6453776	SOL+EDL
10	116201	Rattus norvegicus	chr1	7726222	7726518	1:7726222-7726518	EDL

Figure SD4. List of revealed rat enhancers in GTRD database.

Differentially expressed peaks

We calculated the differentially expressed peaks in two ways: for each experiment phase separately, in comparison with control samples (phase-control peak signatures), and in time course manner comparing the first day of disuse with control samples and each other phase of experiment with the previous phase (time course peak signatures) [35]. Differentially expressed peaks were

annotated with Ensembl gene coordinates and statistics of affected genes ('gene-centered' differentially expressed peaks - gene signatures, DEGs) were calculated.

We counted a gene as differentially expressed if any of its TSS was differentially expressed

A DPI peak was annotated as gene TSS if its center expanded by 200nt intersected the genomic interval of the said gene.

	EDL		Sol	
	UP	DOWN	UP	DOWN
D1vC	1443	774	1035	1423
D3vD1	825	1886	560	373
D7vD3	251	45	136	143
R1vD7	22	150	***19077***	***9198***
R3vR1	32	22	***3603***	***11921***
R7vR3	0	42	34	`47

Table SD4. Differentially expressed peaks, time course (FDR BH < 0.05, |LFC| >1.25)

	EDL		Sol	
	UP	DOWN	UP	DOWN
D1vC	212	145	204	238

D3vD1	162	253	126	78
D7vD3	54	11	20	43
R1vD7	8	35	***2847***	***934***
R3vR1	6	6	***486***	***1490***
R7vR3	0	13	12	9

Table SD5. Differentially expressed peaks, time course gene centric (FDR BH < 0.05, |LFC| >1.25)

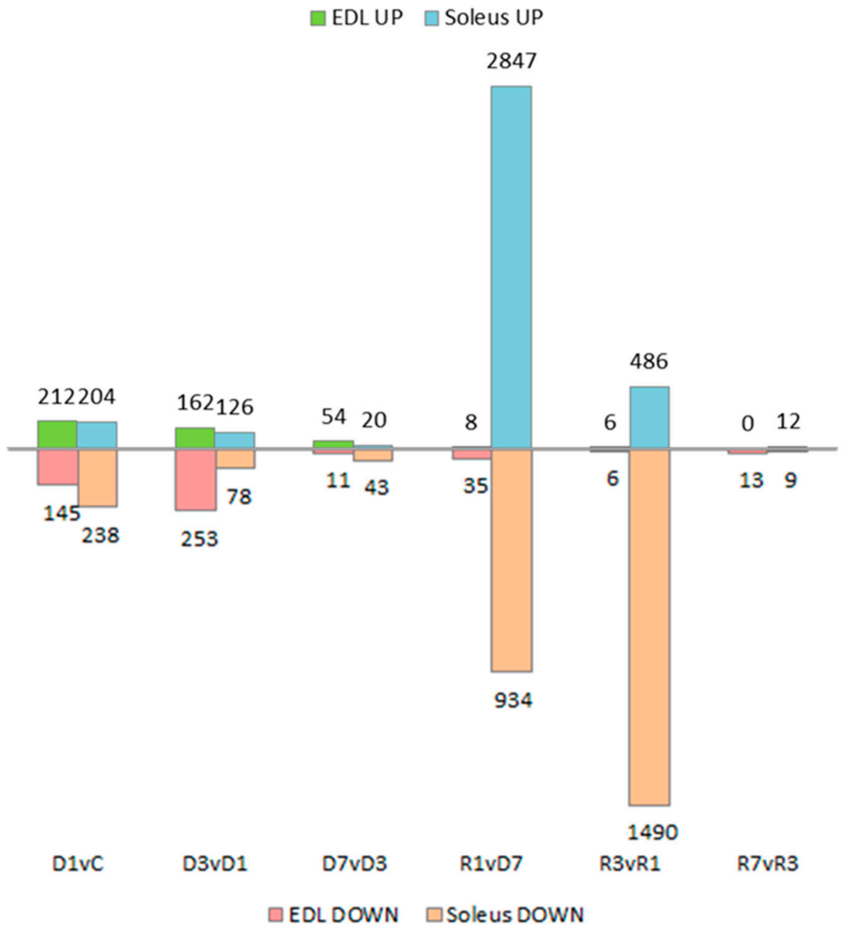

Fig SD5. DEGs in soleus and EDL muscles, time course

	extensor digitorum longus (EDL)		soleus (Sol)	
	UP	*DOWN*	*UP*	*DOWN*
D1	1444	775	1036	1424
D3	1746	1582	594	590

D7	1081	732	525	754
R1	73	180	***18259***	***11689***
R3	517	1270	2344	1916
R7	22	30	1969	734

Table SD6. Differentially expressed peaks, compared to control samples (FDR BH < 0.05, |LFC| >1.25)

	extensor digitorum longus (EDL)		soleus (Sol)	
	UP	*DOWN*	*UP*	*DOWN*
D1	212	145	204	238
D3	282	241	138	80
D7	153	92	101	105
R1	26	10	***2625***	***967***
R3	159	22	572	134
R7	8	7	349	117

Table SD7. Differentially expressed peaks - gene centric, compared to control samples (FDR BH < 0.05, |LFC| >1.25)

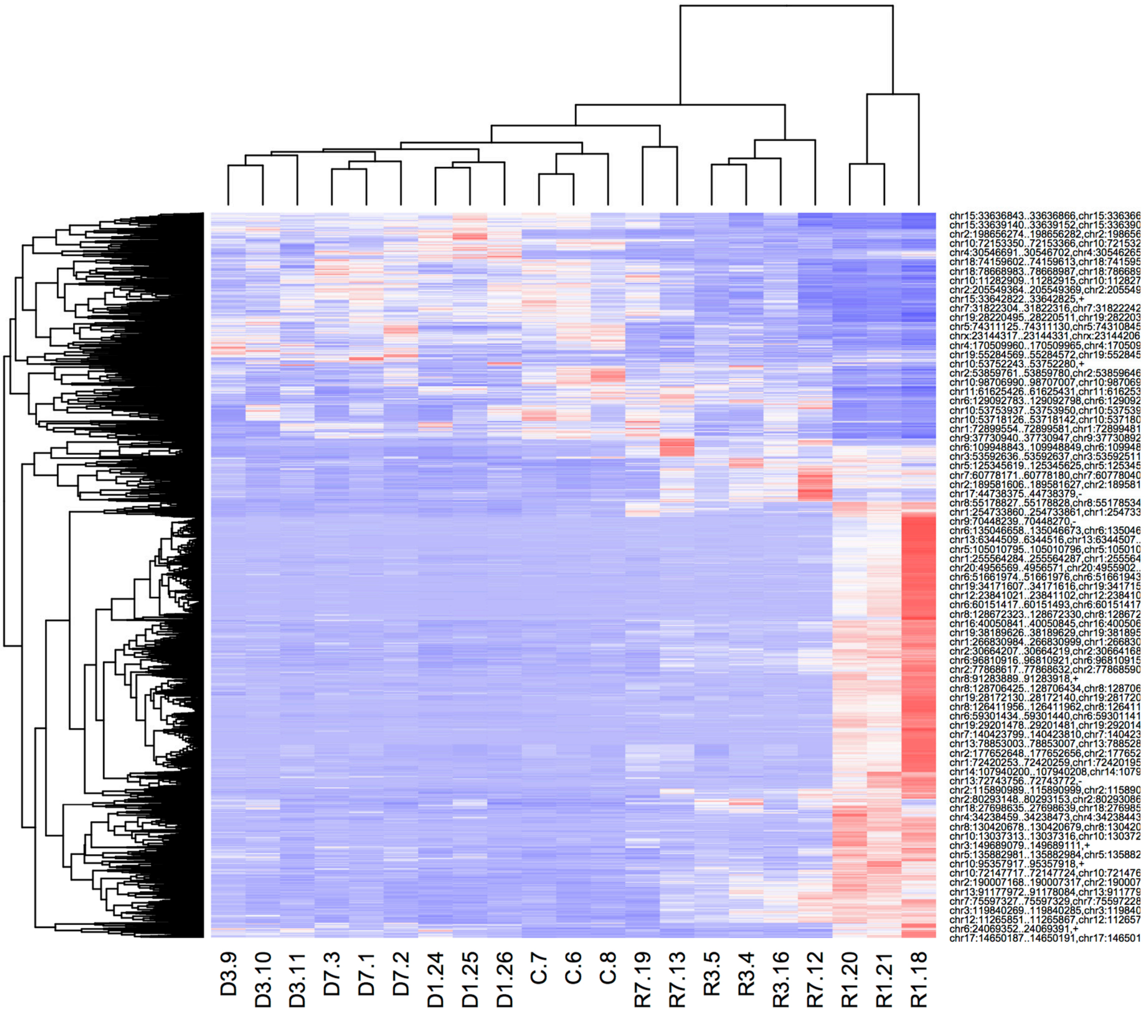

Fig SD6. Differentially expressed peaks (time course) as classifier signatures. FDR threshold 5e-04

EDL

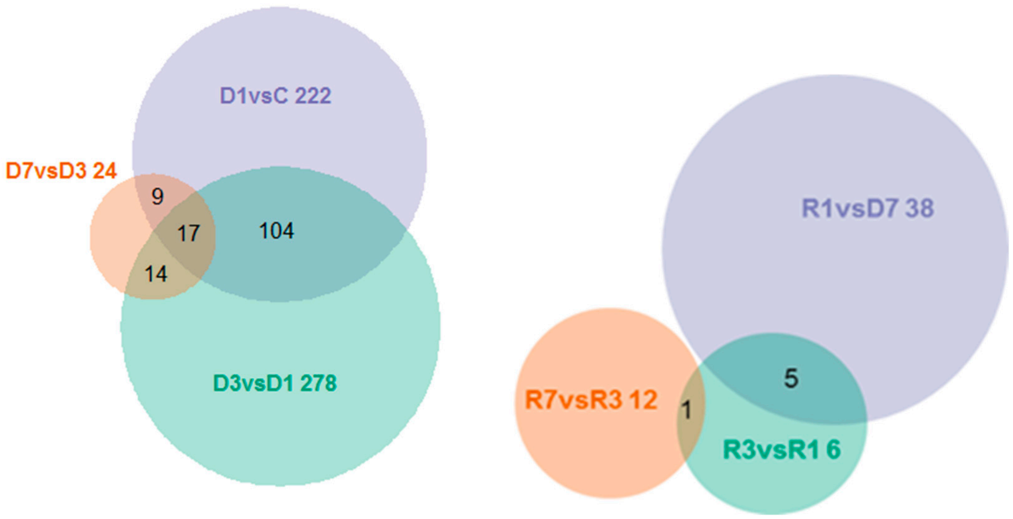

Soleus

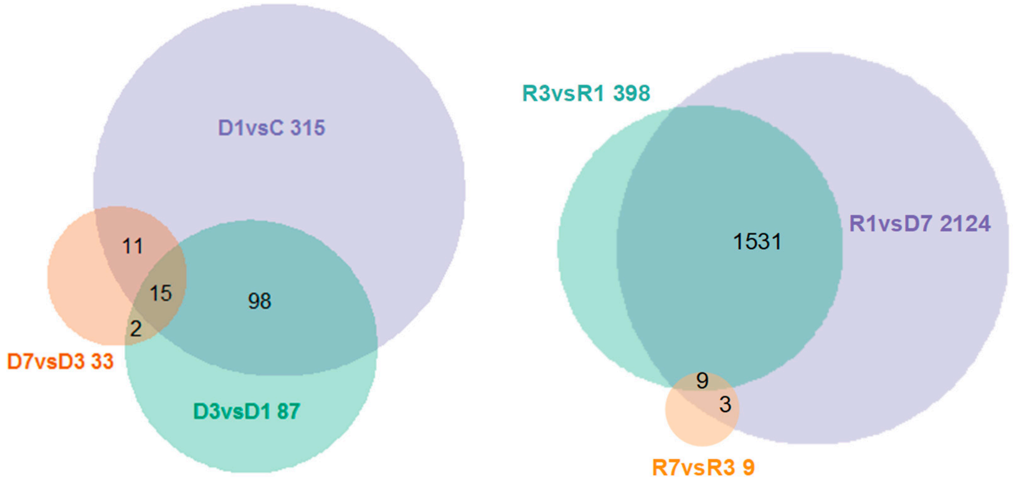

Fig. SD7. Most of the differentially expressed peaks were common to both phase-control and time course comparisons

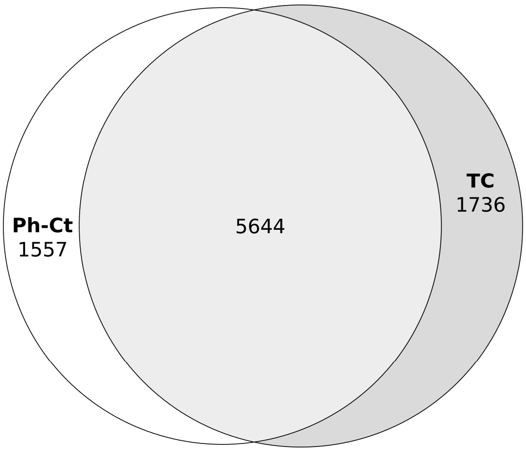

Fig SD8. Common and unique differentially expressed peaks between phase-control (**Ph-Ct**) and time course (**TC**) comparisons. Soleus muscle. FDR threshold 5e-04

**Parent**	**Term**	**D1**	**D3**	**D7**	**R1**	**R3**	**R7**
**actin cytoskeleton organization**	DNA packaging			3.76		5.49	4.53
	chromatin assembly or disassembly			3.95		5.90	6.89
	mitochondrion organization	2.70			5.96		

	cellular component organization	2.71		2.66	5.41	6.67	7.84
	actin cytoskeleton organization	4.02	3.59	2.89	11.61	4.05	3.38
	collagen fibril organization						5.57
	macromolecular complex subunit organization		2.05		3.17	7.61	4.37
	chromosome organization			2.55		5.39	4.51
	protein-DNA complex subunit organization			2.69		5.75	4.25
	supramolecular fiber organization	4.82		4.64	9.47	5.25	6.67
	actin filament-based process	3.90	3.52	2.82	11.32	4.36	3.26
**muscle cell differentiation**	muscle system process			2.81	7.59	2.47	
	muscle contraction			2.84	7.69	2.49	
	cellular component assembly involved in morphogenesis			2.09	5.64	5.82	
	cardiac muscle cell differentiation				3.63	7.71	2.90
	muscle tissue development	2.43			2.62	5.82	2.09
	muscle structure development	2.50			6.79	5.71	2.81
**cellular component organization or biogenesis**	cellular component organization or biogenesis	2.56		2.69	6.44	7.08	7.87
**cellular process**	cellular process				7.29	3.70	3.12
**energy derivation by oxidation of**	purine ribonucleotide metabolic	2.62			10.34		

**organic compounds**	process						
	energy derivation by oxidation of organic compounds				13.60		
	organophosphate metabolic process	3.44			7.80		3.65
	small molecule metabolic process				7.21		3.11
	enzyme linked receptor protein signaling pathway				5.51		
	G-protein coupled receptor signaling pathway	2.66			6.03	2.56	
	oxidation-reduction process				13.05		2.31
**protein folding**	protein folding				5.81		
**mitochondrion organization**	mitochondrion organization				5.96		
**generation of precursor metabolites and energy**	generation of precursor metabolites and energy				13.74		
	phosphorus metabolic process				5.48		2.29
**hydrogen ion transmembrane transport**	hydrogen ion transmembrane transport				9.41		
**T cell mediated immunity**	immune effector process			6.45	2.04		
	T cell mediated immunity	5.58	5.50	10.99	2.92	2.73	3.74

Table SD8. Enriched GO terms of DEGs in soleus muscle. Up-regulated signatures are shown in red, down-regulated - in green (see ST7)

Parent	Term	D1 DOWN	D3 DOWN	D7 DOWN	R1 DOWN	R3 DOWN	R7 DOWN
ATP metabolism	ATP metabolic process		4.66574				
	purine-containing compound metabolic process		2.17223				
T cell mediated immunity	T cell mediated immunity	5.13377					

Table SD9. Enriched GO terms of DEGs, downregulated in EDL muscle

		D1 UP	D3 UP	D7 UP	R1 UP	R3 UP	R7 UP
actin cytoskeleton organization	actin cytoskeleton organization		5.73436				
	supramolecular fiber organization		5.15773				
	actin filament-based process		6.31542				
muscle structure development	cellular component assembly involved in		3.50093		2.05336		

	morphogenesis						
	cellular component morphogenesis		2.50959				
	muscle structure development		6.12048				
	muscle tissue development		2.26282				
	striated muscle cell development		5.54289				
	muscle contraction		5.66158	4.24029	3.38392		
	muscle system process		5.57556	4.17898	3.34631		
**T cell mediated immunity**	leukocyte migration					2.35439	

Table SD10. Enriched GO terms of DEGs, upregulated in EDL muscle. (See ST7)

Gene terms enrichment:

We also found that 34 genes related to both skeletal muscle cell differentiation and positive regulation of transcription by RNA polymerase II had robust DPI peaks significantly upregulated

in disuse phases (See ST11). In the fast muscle most changes happened on the third day of disuse (phase D3) and also included actin cytoskeleton (sarcomere) reorganisation. Nevertheless, ATP metabolism was down-regulated in D3, regardless of the muscle structure development in D3, D7 and R1. T-cell immunity features were down-regulated in D1 but already up-regulated in R3.

We also conducted analagous GSEA for DEGs which were unique in each phase (D1-3-7 and R1-3-7). The number of corresponding DEGs is presented on venn diagrams in Fig.N above (see the list of unique DEGs on each phase and PANTHER results in SM).The analysis was performed using PANTHER GO-Slim Biological Process as an annotation data set via Fisher's test considering the Bonferroni correction for multiple testing. As in the case of general DEGs in each phase, we do observe significant differences in GSEs for unique differentially expressed genes between fast and slow muscles. The regulation of adaptive immune response and related T cell immunity were only enriched terms in the last disuse phase (day 7) in slow muscle, while the regulation of adaptive immune response and ATP, nucleotides metabolic processes were presented in the enrichment set at days 1 and 3 of the disuse in fast muscles, correspondingly. GSEs of the unique DEGs for recovery are distinct between EDL and Sol too. However, the analysis identified that myofibril assembly and muscle contraction are enriched terms in EDL and specific for R1 phase only, whereas no statistically significant terms for unique EDL's differentially expressed genes were revealed in other recovery phases. Interestingly, gene set enrichments in soleus muscle are much widely represented in each recovery phase and demonstrate consistent functional pattern during recovery in this type of the skeletal muscle: involvement of signaling (via G-protein-coupled receptor and phosphorylation cascades of intermediate and target proteins) and metabolic processes (cellular respiration, nucleotides biosynthesis, actin filament organization) in early phase

R1, follow-up activation of translational complex machinery in R3 and cell development and differentiation in R7 phase.

We performed functional analysis on differentially expressed muscle specific peaks which were upstream of a DEG by extrapolating data on functional annotation of those DEGs

In recovery phase the most dispensable of representative biological process ontologies of upregulated muscle specific differentially expressed peaks involved muscle system process and cell differentiation signatures:

term ID	description	frequency	log10 p-value	uniqueness	dispensability
GO:0003012	muscle system process	1.799 %	-8.391	0.96	0
GO:0019058	viral life cycle	0.864 %	-20.793	0.97	0
GO:0070972	protein localization to endoplasmic reticulum	0.218 %	-20.2685	0.94	0
GO:0042026	protein refolding	0.088 %	-5.8876	0.98	0
GO:0016071	mRNA metabolic process	2.522 %	-18.2609	0.86	0
GO:0006457	protein folding	0.935 %	-10.5739	0.99	0

GO:0045597	positive regulation of cell differentiation	4.656 %	-13.8857	0.72	0.01

Table SD11. Representative GO BP terms of DEGs downstream to permissive peaks of soleus muscle upregulated in R1, unique to experiment (REVIGO dispensability value less than 0.05, FDR less than 0.0005). See ST12 for full list of representative terms.

In the same recovery phase, the most dispensable of representative biological process ontologies of genes, associated with down-regulated peaks, which were unique to the experiment, involved muscle system process, metabolism and, interestingly, second-messenger signalling

**term ID**	**description**	**frequency**	**log10 p-value**	**uniqueness**	**dispensability**
GO:0003012	muscle system process	1.799 %	-27.2706	0.93	0
GO:0022904	respiratory electron transport chain	0.435 %	-31.1881	0.68	0
GO:1902600	hydrogen ion transmembrane transport	0.641 %	-11.0666	0.78	0.01
GO:0019932	second-messenger-mediated signaling	1.070 %	-4.5735	0.94	0.02

Table SD12. Representative GO BP terms of downregulated peaks, unique to experiment (REVIGO dispensability value less than 0.05, FDR less than 0.0005). See ST13 for full list of representative terms.

In EDL, muscle specific differentially expressed peaks were mostly related to muscle system process, AMP metabolism, protein signalling, and regulation of immune response along all phases of experiment.

**term ID**	**description**	**frequency**	**log10 p-value**	**uniqueness**	**dispensability**
GO:0003012	muscle system process	1.799 %	-21.1862	0.91	0
GO:0009167	purine ribonucleoside monophosphate metabolic process	1.382 %	-14.1246	0.43	0
GO:0007167	enzyme linked receptor protein signaling pathway	3.968 %	-4.3663	0.92	0.02
GO:0002697	regulation of immune effector process	1.323 %	-3.9525	0.95	0.05

PANTHER GO-Slim Biological Process	Reference	Experiment	expected	Fold Enrichment	P value
nucleosome organization	50	8	0.89	8.96	1.27E-02
chromatin organization	250	17	4.46	3.81	9.34E-03
chromosome organization	379	20	6.77	2.96	4.92E-02
protein-DNA complex subunit organization	106	11	1.89	5.81	1.18E-02
protein-containing complex subunit organization	543	27	9.69	2.79	5.77E-03
actin cytoskeleton organization	240	16	4.28	3.73	2.22E-02
actin filament-based process	248	16	4.43	3.61	3.24E-02
cellular component biogenesis	969	37	17.3	2.14	3.66E-02

Table SD13. Significantly enriched features in DEGs, having a robust non-genic signature upstream TSS peak (See ST9). Bonferroni corrected P value was cut off at $P < 0.05$.

Enhancers

	EDL		Sol	
	UP	DOWN	UP	DOWN
D1	15	8	12	29
D3	15	28	5	4
D7	9	7	6	11
R1	0	1	407	121
R3	7	3	19	11
R7	0	0	21	6
Total	33	40	437	146

Table SD14. Differentially expressed enhancers in soleus and EDL muscle

In Soleus at R1 phase differentially expressed enhancers formed clear clusters, unlike the other phases

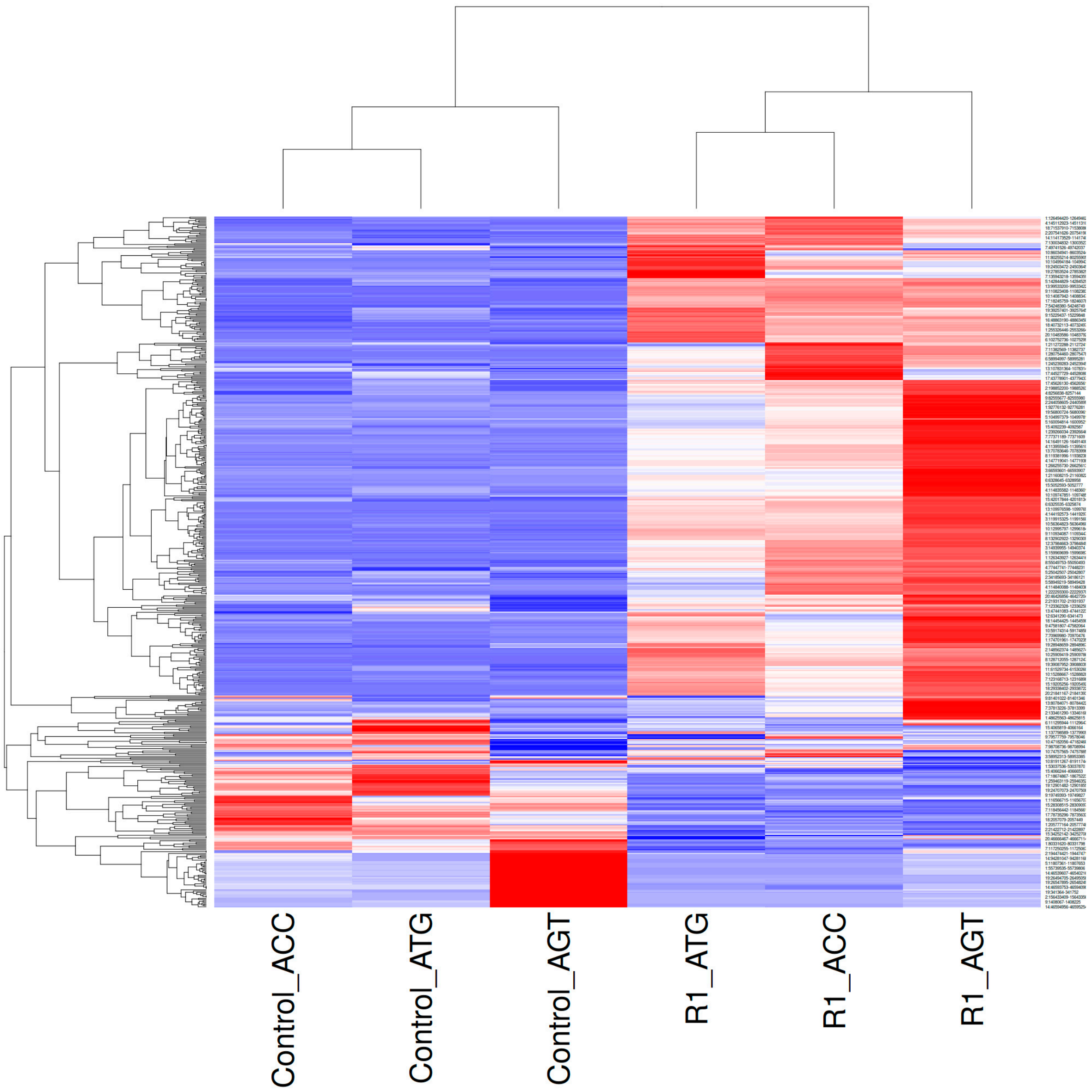

Fig. SD9. Heatmap of differential expression of enhancers in the Soleus muscle on the first day of recovery (R1 phase)

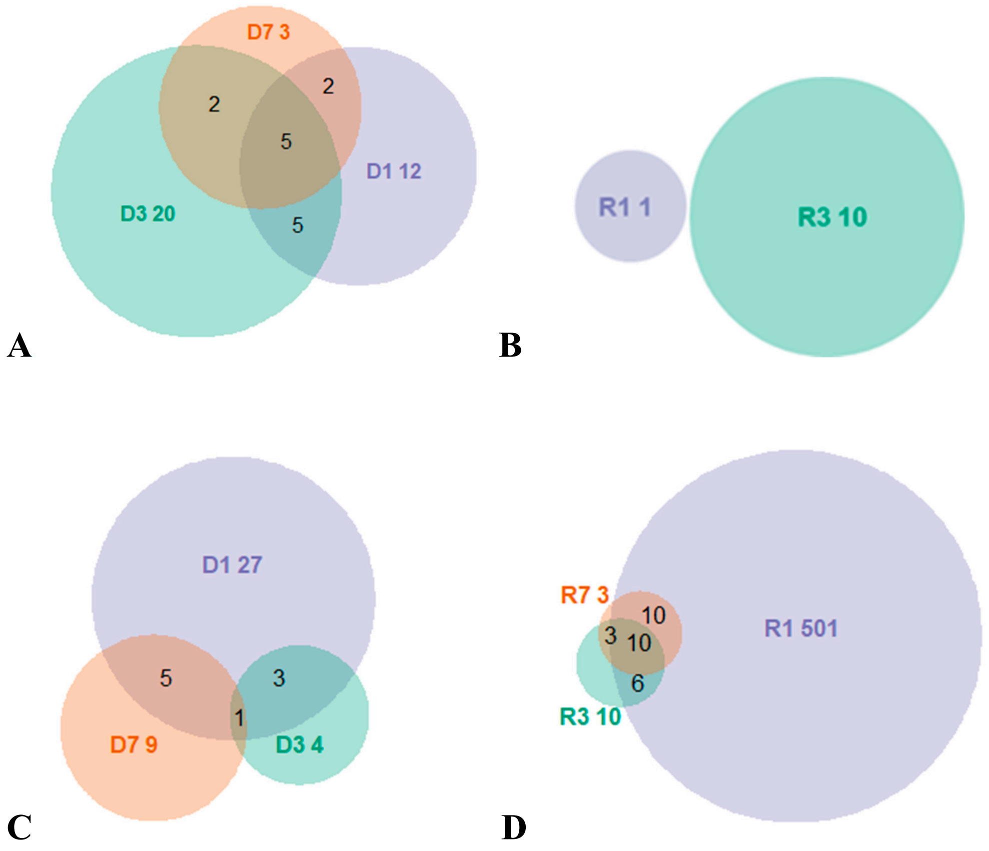

Fig. SD10. Venn diagrams for differentially expressed enhancers. **A**. Differentially expressed enhancers on days 1, 3 and 7 of disuse in the EDL muscle (shown as D1, D3, D7 respectively). **B**. Differentially expressed enhancers on days 1, 3 and 7 of recovery in the EDL muscle (shown as R1, R3, R7 respectively). **C**. Differentially expressed enhancers on days 1, 3 and 7 of disuse in the soleus muscle (shown as D1, D3, D7 respectively). **D**. Differentially expressed enhancers on days 1, 3 and 7 of recovery in the soleus muscle (shown as R1, R3, R7 respectively).

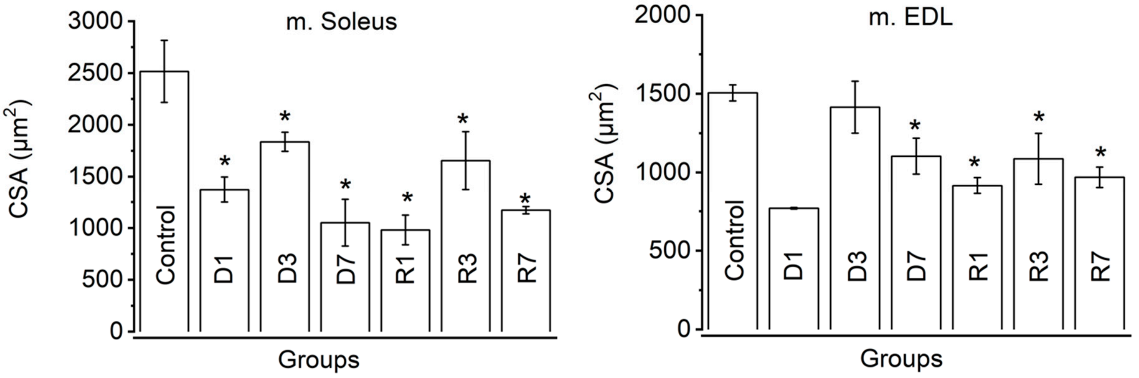

Fig. SD11 Cross-sectional area values in control and time-point groups in slow and fast muscle.
