# Supplementary material for: Genome-Wide Atlas of Promoter Expression Reveals Contribution of Transcribed Regulatory Elements to Genetic Control of Disuse-Mediated Atrophy of Skeletal Muscle
Source: Biology (Basel). 2021 Jun 20;10(6):557. doi: 10.3390/biology10060557 (PMC8235325; doi:10.3390/biology10060557)
Supplement: Supplementary file 1 [file biology-10-00557-s001.zip › SM.zip!/PANTHER - Compare lists to reference list_D3_unique.html]

PANTHER - Compare lists to reference list


Login     
  Register    
  Contact us

|  |  |  |  |  |  |  |  |  |  |  |  |  |  |  |  |
| --- | --- | --- | --- | --- | --- | --- | --- | --- | --- | --- | --- | --- | --- | --- | --- |
|  | Home |  | About |  | PANTHER Data |  | PANTHER Tools |  | PANTHER Services |  | Workspace |  | Downloads |  | Help/Tutorial |

|  |  |
| --- | --- |
|  | **PANTHER will be unavailable on December 18th, 2020 for maintenance starting at 10:00AM PST** |

Analysis Summary: Please report in publication 


|  |  |  |
| --- | --- | --- |
| **Analysis Type:** PANTHER Overrepresentation Test (Released 20200728) |  |  |
||  |
| --- |
| --- |
 **Annotation Version and Release Date:** PANTHER version 15.0 Released 2020-02-14 |  |  |
| --- |  |  |
| **Analyzed List:** \| EDL D3\_exclusively\_315 genes.txt (Rattus norvegicus) \| Change; --- \| \|; **Reference List:** \| Rattus norvegicus (all genes in database) \| Change | |  |
| --- |  |  |
| **Annotation Data Set:**    GO biological process complete  GO molecular function complete  GO cellular component complete  PANTHER Pathways  PANTHER GO-Slim Molecular Function  PANTHER GO-Slim Biological Process  PANTHER GO-Slim Cellular Component  PANTHER Protein Class  Reactome pathways | |  |
| --- |  |  |
| **Test Type:**  Fisher's Exact   Binomial | | |
| --- |  |  |
| **Correction:**  Calculate False Discovery Rate   Use the Bonferroni correction for multiple testing     No correction | | |

  
Results

  


|  |
|  |
|

|  |  |  |
| --- | --- | --- |
|  | Reference list | EDL D3\_exclusively\_315 genes.txt |
| Uniquely Mapped IDS: | 21677 out of 21677 | 268 out of 319 |
| Unmapped IDs: | 0 | 47 |
| Multiple mapping information: | 0 | 24 |

  

Bonferroni count: 1705

Export  Table  XML with user input ids  JSON with user input ids
  View:
-- Please select a chart to display --
Multiple Pie Chart
Bar Chart of Gene Count
Overlaid Area Chart of Difference
Bar Chart of Difference
  
  
Displaying only results for Bonferroni-corrected for P < 0.05, click here to display all results
  
  

|  |  |  |  |  |  |  |  |
| --- | --- | --- | --- | --- | --- | --- | --- |
|  | Rattus norvegicus (REF) | EDL D3\_exclusively\_315 genes.txt ( Hierarchy  NEW! ) | | | | | |
| PANTHER GO-Slim Biological Process | # | # | expected | Fold Enrichment | +/- | P value |
| ATP metabolic process | 66 | 10 | .97 | 10.30 | + | 2.50E-04 |
| purine ribonucleotide metabolic process | 137 | 12 | 2.02 | 5.95 | + | 3.24E-03 |
| purine nucleotide metabolic process | 146 | 12 | 2.15 | 5.59 | + | 5.98E-03 |
| purine-containing compound metabolic process | 161 | 12 | 2.37 | 5.06 | + | 1.52E-02 |
| nucleotide metabolic process | 173 | 12 | 2.55 | 4.71 | + | 2.99E-02 |
| nucleoside phosphate metabolic process | 178 | 12 | 2.62 | 4.58 | + | 3.90E-02 |
| ribonucleotide metabolic process | 142 | 12 | 2.09 | 5.74 | + | 4.58E-03 |
| ribose phosphate metabolic process | 151 | 12 | 2.22 | 5.40 | + | 8.26E-03 |
| Unclassified | 12357 | 168 | 181.85 | .92 | - | 0.00E00 |

  
  


About   |  
Release Information   |  
Contact Us   |  
System Requirements   |  
Privacy Policy   |  
Disclaimer
  
© Copyright 2020 Paul Thomas All Rights Reserved.
