# Supplementary material for: Genome-Wide Atlas of Promoter Expression Reveals Contribution of Transcribed Regulatory Elements to Genetic Control of Disuse-Mediated Atrophy of Skeletal Muscle
Source: Biology (Basel). 2021 Jun 20;10(6):557. doi: 10.3390/biology10060557 (PMC8235325; doi:10.3390/biology10060557)
Supplement: Supplementary file 1 [file biology-10-00557-s001.zip › SM.zip!/PANTHER - Compare lists to reference list_R1_unique.html]

PANTHER - Compare lists to reference list


Login     
  Register    
  Contact us

|  |  |  |  |  |  |  |  |  |  |  |  |  |  |  |  |
| --- | --- | --- | --- | --- | --- | --- | --- | --- | --- | --- | --- | --- | --- | --- | --- |
|  | Home |  | About |  | PANTHER Data |  | PANTHER Tools |  | PANTHER Services |  | Workspace |  | Downloads |  | Help/Tutorial |

|  |  |
| --- | --- |
|  | **PANTHER will be unavailable on December 18th, 2020 for maintenance starting at 10:00AM PST** |

Analysis Summary: Please report in publication 


|  |  |  |
| --- | --- | --- |
| **Analysis Type:** PANTHER Overrepresentation Test (Released 20200728) |  |  |
||  |
| --- |
| --- |
 **Annotation Version and Release Date:** PANTHER version 15.0 Released 2020-02-14 |  |  |
| --- |  |  |
| **Analyzed List:** \| EDL R1\_exclusively\_25 genes.txt (Rattus norvegicus) \| Change; --- \| \|; **Reference List:** \| Rattus norvegicus (all genes in database) \| Change | |  |
| --- |  |  |
| **Annotation Data Set:**    GO biological process complete  GO molecular function complete  GO cellular component complete  PANTHER Pathways  PANTHER GO-Slim Molecular Function  PANTHER GO-Slim Biological Process  PANTHER GO-Slim Cellular Component  PANTHER Protein Class  Reactome pathways | |  |
| --- |  |  |
| **Test Type:**  Fisher's Exact   Binomial | | |
| --- |  |  |
| **Correction:**  Calculate False Discovery Rate   Use the Bonferroni correction for multiple testing     No correction | | |

  
Results

  


|  |
|  |
|

|  |  |  |
| --- | --- | --- |
|  | Reference list | EDL R1\_exclusively\_25 genes.txt |
| Uniquely Mapped IDS: | 21677 out of 21677 | 23 out of 23 |
| Unmapped IDs: | 0 | 2 |
| Multiple mapping information: | 0 | 0 |

  

Bonferroni count: 1705

Export  Table  XML with user input ids  JSON with user input ids
  View:
-- Please select a chart to display --
Multiple Pie Chart
Bar Chart of Gene Count
Overlaid Area Chart of Difference
Bar Chart of Difference
  
  
Displaying only results for Bonferroni-corrected for P < 0.05, click here to display all results
  
  

|  |  |  |  |  |  |  |  |
| --- | --- | --- | --- | --- | --- | --- | --- |
|  | Rattus norvegicus (REF) | EDL R1\_exclusively\_25 genes.txt ( Hierarchy  NEW! ) | | | | | |
| PANTHER GO-Slim Biological Process | # | # | expected | Fold Enrichment | +/- | P value |
| myofibril assembly | 23 | 3 | .02 | > 100 | + | 4.54E-03 |
| cellular component assembly involved in morphogenesis | 26 | 3 | .03 | > 100 | + | 6.36E-03 |
| striated muscle cell development | 31 | 3 | .03 | 91.21 | + | 1.04E-02 |
| striated muscle cell differentiation | 35 | 3 | .04 | 80.78 | + | 1.46E-02 |
| muscle cell differentiation | 42 | 3 | .04 | 67.32 | + | 2.44E-02 |
| muscle structure development | 52 | 3 | .06 | 54.37 | + | 4.49E-02 |
| muscle cell development | 33 | 3 | .04 | 85.68 | + | 1.24E-02 |
| actomyosin structure organization | 51 | 3 | .05 | 55.44 | + | 4.25E-02 |
| muscle contraction | 43 | 4 | .05 | 87.67 | + | 2.83E-04 |
| muscle system process | 44 | 4 | .05 | 85.68 | + | 3.08E-04 |
| Unclassified | 12357 | 9 | 13.11 | .69 | - | 0.00E00 |

  
  


About   |  
Release Information   |  
Contact Us   |  
System Requirements   |  
Privacy Policy   |  
Disclaimer
  
© Copyright 2020 Paul Thomas All Rights Reserved.
